# The hydrocarbon-degrading bacteria and fungi in oil contaminated soils of Kazakhstan: microbiome composition, enrichment, isolation and bioremediation potential

**DOI:** 10.1186/s40793-026-00866-y

**Published:** 2026-03-07

**Authors:** Felix Müller, Haitao Wang, Anne Reinhard, Anel Omirbekova, Ramza Berzhanova, Togzhan Mukasheva, Tim Urich, Annett Mikolasch

**Affiliations:** 1 Institute of Microbiology, University Greifswald, Felix-Hausdorff-Straße 8, 17489 Greifswald, Germany; 2 Department of Biology and Biotechnology, Al-Farabi Kazakh National University, Al-Farabi Ave 71, Almaty, 050040 Kazakhstan

**Keywords:** Oil degradation, Crude oil, Microbiome, *Meyerozyma guilliermondii*, Prokaryotes, Eukaryotes, Enrichment, Isolation

## Abstract

**Background:**

Oil contamination in soils causes significant environmental impacts and risks to human health. Oil components can be naturally reduced by indigenous microorganisms, that are able to degrade such substrates. We used culture-independent and culture-dependent methods to examine the prokaryotic and the eukaryotic microbiome of different heavily oil contaminated soils in Kazakhstan. Bacteria and fungi were enriched and isolated from four soils contaminated with crude oil or hydrocarbons. Aliphatic, aromatic and condensed aromatic model hydrocarbons of crude oil and crude oil itself were used as substrates for the enrichment and the isolation experiments. The enrichment process was accompanied by culture-independent tests.

**Results:**

The results of the Illumina sequencing of the contaminated soils and the enrichment cultures were compared with the results of the culture-dependent isolation and determination of bacterial, yeast and filamentous fungal strains. The majority of these 110 strains from 45 different genera belong to well-described hydrocarbon degraders like Bacilli and Rhodococci as well as to *Achromobacter*,* Gordonia*,* Pseudomonas*, *Stenotrophomonas*, *Aspergillus*,* Exophiala*,* Fusarium*,* Meyerozyma*,* Penicillium* and *Trichoderma* species. The most abundant species was the ascomycetal yeast *Meyerozyma guilliermondii* followed by strains of the bacterial genus *Peribacillus*. Furthermore, we combined the microbiome insights on the enrichment procedures and the isolation of bacteria, yeasts and filamentous fungi with the in-fact degradation potential of the isolated species based on substrate consumption and metabolite formation. In addition to the well-described hydrocarbon degraders, the utilization spectrum of less-studied strains of the genera *Leifsonia*, *Neorhizobium*, *Purpureocillium*, *Rhodotorula* and *Sarocladium* could be broadened.

**Conclusion:**

In the end a complex overview of the indigenous microorganisms and their degradation ability of crude oil components emerged and demonstrates the great potential of bioremediation for Kazakhstan soils.

**Supplementary Information:**

The online version contains supplementary material available at 10.1186/s40793-026-00866-y.

## Background

 Soil contamination by crude oil and oil hydrocarbons has been a global environmental problem for decades [1–5]. Pollution of water and soil by crude oil is the result of explosions, production, transportation, storage and accidental releases of oil and causes significant environmental impacts and risks to human health [6–10]. Upstream oil extraction to transport the crude oil to the surface has potential health impacts in communities near oil extraction such as liver damages, immunodeficiency, cancer and neurological symptoms [11]. Components of crude oil such as polycyclic aromatic hydrocarbons (PAH) are described as toxic, carcinogenic and mutagenic pollutants and have a negative impact on the biological properties of soils [8, 12–14]. PAHs absorb into root surfaces after crude oil contamination of soil and also into the leaves after volatilization from the soil [15] and thus they permeate the food webs. The downward movements of crude oil components can also pollute the groundwater, an important drinking water resource, and can therefore pose risks to human health and the environment [6, 12].

Numerous studies have demonstrated that degradation of crude oil components by bacteria and fungi is the most important natural biological factor involved in bioremediation of oil pollutants [16–21]. Using indigenous or artificially added microorganisms with high degradation potential bioremediation of soils can be achieved by natural attenuation, biostimulation, and bioaugmentation. Microbial species that degrade different hydrocarbons were isolated and studied over decades with regard to their degradation pathways and efficiency [22–29]. These investigations provided the basics for annotating extensive metagenomic datasets, helped to identify enzymes and organisms that were useful in bioremediation processes, but these culture-dependent methods only cover a very small range of the diversity of their natural habitats [30]. With the emergence and advancement in sequencing techniques, microbiomes in contaminated habitats such as soils have been investigated using culture-independent methods. Mostly the bacterial microbiome was examined [31–41]. Much less frequently, these studies were combined with those of the fungal microbiome [42–46]. Even less frequently, investigations of the microbiome in contaminated soils were combined with the enrichment and isolation of hydrocarbon-degrading microorganisms of bacterial and fungal origin using both culture-independent and culture-dependent methods [2, 30].

In this study, we used both culture-independent and culture-dependent methods to examine the prokaryotic and the eukaryotic microbiome of different heavily contaminated soils from oil deposit in Kazakhstan. Using the culture-dependent approach, we enriched and isolated microorganisms with the ability to degrade crude oil components from the contaminated soils. With the culture-independent approach, 16 S rRNA and 18 S rRNA gene amplicon sequencings were employed to identify the microbiomes in the enrichment cultures of bacteria and fungi, respectively. To enhance the number of bacterial and fungal isolates we used crude oil and eight different model compounds of crude oil as isolation substrates: tetradecane as model for *n*-alkanes, pristane and heptamethylnonane for branched chain alkanes, cyclohexanone for alicyclic hydrocarbons, phenol and biphenyl for low molecular weight aromatics and anthracene as a simple model of PAHs. After isolation and taxonomic characterisation of the isolated bacteria and fungi their degradation potential of the isolation substrates was determined and assessed with regard to bioremediation.

## Materials and methods

### Sample sites

In the middle of January 2018, two oil-contaminated soil samples were taken from the Uzen oil deposit (B1 and B2–43°26′36″ N 52°46′56″ E). The residual hydrocarbon contents (mg/kg soil) were different in the soils B1 and B2, B1 60,000–80,000 mg/kg soil, B2 100,000–250,000 mg/kg soil. Another oil-contaminated soil sample was taken at the end of April from the Aktobe oil deposit in the Kenkiyak region (B3–50°20’38.2” N 57°05’11.0” E, residual hydrocarbon contents (mg/kg soil) 10000–30000). In the middle of April 2018, a control soil sample was taken from a park close by the Almaty-1 train station, Almaty (BK − 43°20’36.8” N 76°56’30.7” E, residual hydrocarbon contents (mg/kg soil) 500–1000). Further characteristics of the soil samples and the crude oils are listed in the Additional file 1, Table S1.

### Media

Nutrient broth II (pH 6,8; SIFIN, Berlin) served as cultivation medium (pH 6,8; SIFIN, Berlin) for bacteria and medium based on biomalt (pH 5,5; Villa Natura Gesundprodukte, Germany), agar agar powder and water for yeasts and filamentous fungi.

For the isolation of mainly bacteria a mineral salt medium (MSMB, pH 6,3) containing NH_4_H_2_PO_4_ 5 g L^− 1^, K_2_HPO_4_ 2.5 g L^− 1^, MgSO_4_ × 7 H_2_O 0.5 g L^− 1^, NaCl 0.5 g L^− 1^, K_2_SO_4_ 0.46 g L^− 1^, CaCl_2_ 70 mg L^− 1^, FeCl_3_ × 6 H_2_O 2 g L^− 1^ was used. For the isolation of yeasts and filamentous fungi a mineral salt medium (MSMF, pH 5,4) with the compounds NH_4_H_2_PO_4_ 5 g L^− 1^, KH_2_PO_4_ 2.5 g L^− 1^, MgSO_4_ × 7 H_2_O 1 g L^− 1^, Ca(NO_3_)_2_ × 4 H_2_O 20 mg L^− 1^, FeCl_3_ × 6 H_2_O 2 mg L^− 1^ was used. Before the sterilization following supplements were added to the media H_3_BO_3_ 5 g L^− 1^, MnSO_4_ × 5 H_2_O 1 g L^− 1^, ZnSO_4_ × 7 H_2_O 1 g L^− 1^, Na_2_MoO_4_ 4 g L^− 1^, CuSO_4_ × 4 H_2_O 4 g L^− 1^, CoCl_2_ 2 g L^− 1^, KJ 1 g L^− 1^.

1% of a vitamin solution containing myo-Inositol 2 g L^− 1^, thiamine hydrochloride 1 g L^− 1^, nicotinic acid 0.4 g L^− 1^, pantothenic acid 0.4 g L^− 1^, pyridoxine hydrochloride 0.4 g L^− 1^, aminobenzoic acid 0.2 g L^− 1^, riboflavin 0.2 g L^− 1^, biotin 2 mg L^− 1^, folic acid 2 mg L^− 1^ was added to the MSMF before the inoculation.

### Enrichment and isolation of microorganisms

Soil samples (2 g each) were put into round-bottomed flasks containing 100 ml of either MSMB or MSMF. The substrates crude oil (1%, from the Uzen oil deposit), tetradecane (1%), heptamethylnonane (1%), pristane (0.5%) and cyclohexanone (0.25%) were directly added via pipetting. Phenol (0.05%) and anthracene (0.25%) were put into the flasks containing medium and shaken at 180 rpm/30°C at least one night before inoculation. 0.02% yeast extract was added to the first passage of phenol (0.05%) enrichment cultures and 50 mg of phenol added every two days. For the substrate biphenyl (0.25%), a stock solution with diethyl ether was added into the empty flask under a fume hood. After one night of evaporation, the medium was added and the flask and shaken at 180 rpm/30°C at least one night before inoculation.

For the soil samples B3 and BK oil from the Aktobe oil deposit was used. The concentration of biphenyl and anthracene were changed to 0.125%.

After the addition of the soil samples, the flasks were shaken until a strong density was visible for ca. 3 days. 5 ml of culture were used as inoculum for the new flask containing medium and substrate, whereas the rest was decanted into two 50 ml tubes and frozen until extraction of DNA. The length of incubation of each passage varied but never exceeded 2 weeks. Cultures that contained aggregates were homogenized with a sterilized SilentCrusher M (Heidolph Instruments GmbH & CO. KG, Germany). This procedure was repeated until a maximum of 6 times/passages. From the last passage, 100 µl were plated out on rich media plates.

### Microbiome analysis

DNA was extracted from soil samples and cultivation medium of selected points of the enrichment using the DNeasy PowerSoil Kit (QIAGEN, Hilden, Germany). The extracted DNA samples were sent to LGC Genomics (Berlin, Germany) for sequencing. 16 S rRNA and 18 S rRNA genes were amplified with primer-pairs 515YF (GTG YCA GCM GCC GCG GTA A) /B806R (GGA CTA CNV GGG TWT CTA AT) [47] and 1183 F (AATTTGACTCAACRCGGG) /1443R (GRGCATCACAGACCTG) [48] for prokaryotes and eukaryotes, respectively. Amplicons were sequenced with Illumina MiSeq platform for 300 bp paired-end reads. All the sequencing data were deposited to the European Nucleotide Archive of EMBL (European Molecular Biology Laboratory) with the study accession number PRJEB87402.

The amplicon sequencing data were processed using *dada2* pipeline [49] in R v.3.5.0 (R Foundation for Statistical Computing, Austria) as described previously [50]. Briefly, raw sequences were quality checked and filtered, then amplicon sequencing variants (ASVs) were inferred. After removing chimeras, the representative sequence of each ASV was assigned to taxonomy against SILVA v132 [51]. ASVs of 16 S rRNA gene that were assigned as chloroplast or mitochondria were removed.

The alpha-diversity was calculated using the *phyloseq* package [52]. The taxa distribution was shown using bubble plots with relative abundance. The plots were created using *ggplot2* package [53]. BLASTN was used to find the matches between the amplicon sequences and the rRNA sequences obtained from the isolated strains.

### Identification of isolated microorganisms

For the determination of cell morphology (shape, size) of the isolates, a phase-contrast microscope (Axiolab, Zeiss) was used. The KOH test [54] was used to determine the Gram characteristics.

Chromosomal DNA of the strains were isolated by DNeasy PowerSoil-Kit (QIAGEN, Germany) following the manufacturer instructions, using a FastPrep-24 5G (MP Biomedicals, USA) for 45 s with a velocity of 5 m sec^− 1^ for crushing the cells.

The isolated DNA was characterized for prokaryotes by direct determination of the nucleotide sequence of the 16 S rRNA gene fragment and for eukaryotes by internal transcribed spacer (ITS) gene sequence and 18 S region as described previously [55]. The PCR reaction for the 16 S rRNA was performed with universal primers F-5′-AGAGTTTGATYMTGGCTC [56] and the R-5′GGTTACCTTGTTACGACTT [57], for the ITS region with ITS1 F-5’TCCGTAGGTGAACCTGCGG and ITS4 R-5’TCCTCCGCTTATTGATATGC [58] and the 18 S rRNA with the primers F-5’AATTTGACTCAACRCGGG [59] and R-5’GRGCATCACAGACCTG [48].

Sanger sequencing was performed by Eurofins Genomics (Konstanz, Germany) with the named primers. The resulting forward and reverse sequences were assembled using the program Geneious (Geneious, Boston, MA, USA). The sequences were compared with the NCBI databases using the Basic Local Alignment Search Tool (BLAST) algorithm (https://www.ncbi.nlm.nih.gov/, accessed in 2024) [60].

### Metabolic experiments

Pre-cultivated cells of the isolated bacterial and fungal strains were cultivated on the isolation substrates as described previously [55]. Assays without substrates and without cells were used as controls. As sole source of carbon and energy 25 µL of the isolation substrates tetradecane, pristane, heptamethylnonane and cyclohexanone were used in 10 mL assays (substrate concentration 0.25% v/v). Phenol, biphenyl and anthracene were used as substrates according to the modified method described by Sietmann et al. [61] at a final concentration of 0.05% (phenol) and 0.25% (biphenyl, anthracene).

After 5 (for bacteria and yeasts) and 7 (for filamentous fungi) days of incubation the culture of each transformation assay was extracted according to Mikolasch et al. [62]. The extracts obtained were analyzed by high-performance liquid chromatography (HPLC) and gas chromatography–mass spectrometry (GC-MS) [55, 63].

## Results

In total 60 different enrichment cultures were set up from the oil-contaminated soil samples B1, B2, B3, and exhaust contaminated soil sample BK as microbe sources, and using the substrates crude oil, tetradecane, heptamethylnonane, pristane, cyclohexanone, phenol, biphenyl and anthracene as the sole carbon source (Additional file 1: Table S2). During the enrichment, 3–6 subcultures were prepared depending on the turbidity level of the cultures, which were then analysed by microbiome study for their microbial communities and from which hydrocarbon-degrading bacteria, yeasts, and filamentous fungi were also isolated. B2 was not analysed by microbiome study because sufficient DNA could not be obtained. Nevertheless, B2 was used for enrichment approaches and strains were isolated from these enrichment cultures.

### Molecular analyses of prokaryotic and eukaryotic communities

#### Changes in prokaryotic microbiome composition during enrichment culture

Approximately 3.8 million raw reads were generated for all prokaryotic samples. About 3.4 million high-quality reads were retained after pair-end read assembly, quality filtering and chimera removal. Eventually, 2302 ASVs were selected for downstream analyses.

**Fig. 1:**
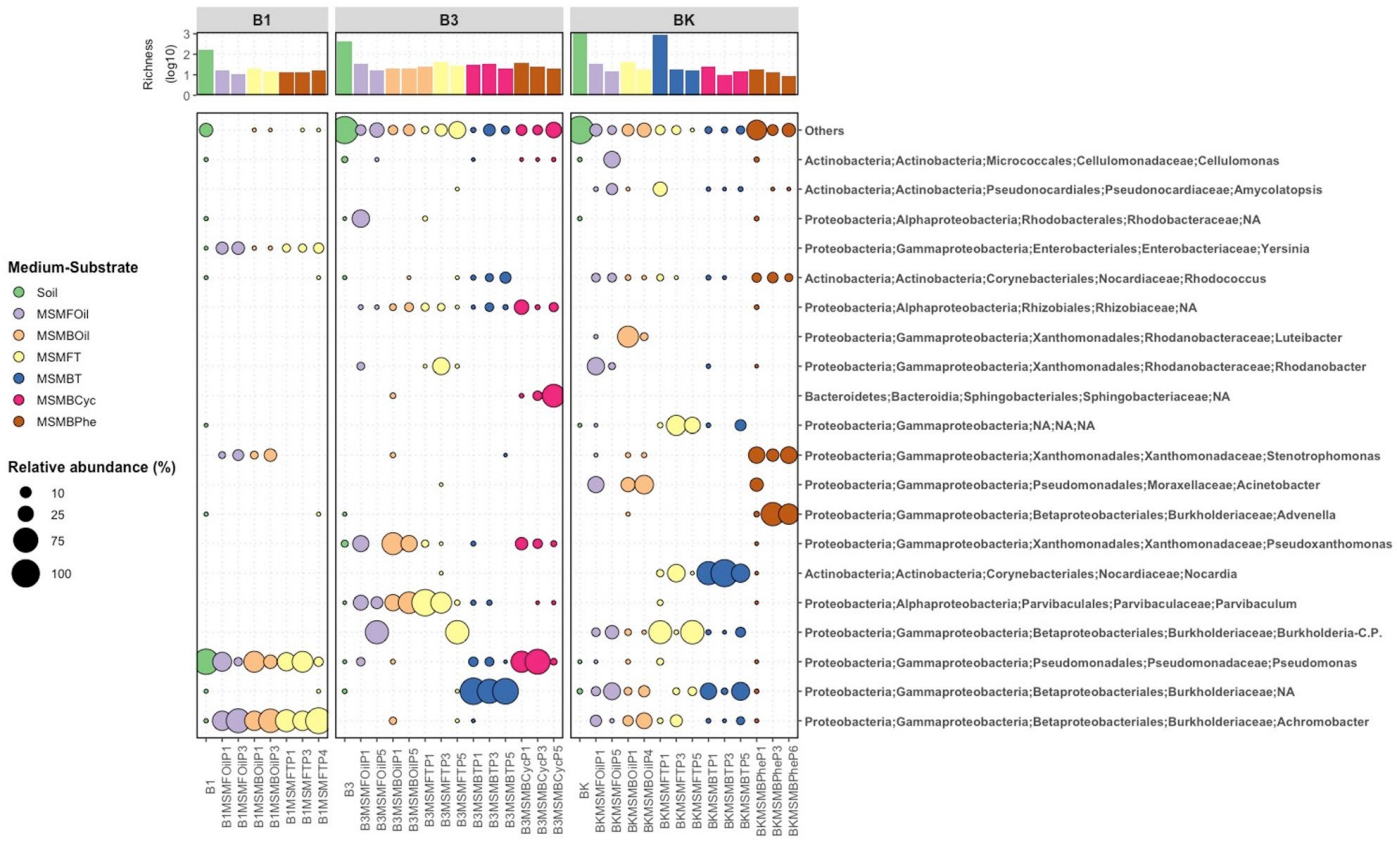
Changes in prokaryotic richness and microbiome composition during enrichment cultures. Soil: original soil sample; MSMFOil: MSMF—mineral salt medium for the isolation of yeasts and fungi, Oil–isolation substrate crude oil; MSMBOil: MSMB—mineral salt medium for the isolation of bacteria, Oil–isolation substrate crude oil; MSMFT: MSMF—mineral salt medium for the isolation of yeasts and fungi, T—isolation substrate tetradecane; MSMBT: MSMB—mineral salt medium for the isolation of bacteria, T—isolation substrate tetradecane; MSMBCyc: MSMB—mineral salt medium for the isolation of bacteria, Cyc—isolation substrate cyclohexanone; MSMBPhe: MSMB—mineral salt medium for the isolation of bacteria, Phe—isolation substrate phenol

Richness (number of ASVs) and alpha diversity of prokaryotes of the soil samples B1, B2, B3 and BK depended from the contamination level and were therefore not detectable in soil sample B2 with the highest residual hydrocarbon content and were highest in the control sample BK, which has the lowest value of the residual hydrocarbons (Fig. 1; Table 1).

**Table 1:** Alpha diversity of prokaryotes versus hydrocarbon contents, salinity and pH of contaminated soils in Kazakhstan

Samples	Chao 1	ASV (Richness)	Shannon	Simpson	Residual hydrocarbon contents (mg/kg soi)	Soil salinity (%)	Soil pH
B1	160	160	2.26	0.79	60,000–80,000	3–5	8.5
B2	–^a^	–	–	–	100,000–250,000	3–5	8.5
B3	425	425	4.09	0.95	10,000–30,000	7–9	9.0-9.3
BK	1219	1219	6.10	0.99	500–1000	0.5	5.1

^a^Soil sample was not analyzed because sufficient DNA could not be obtained

A correlation between the richness and alpha diversity of prokaryotes and soil salinity and acidity could only be demonstrated to a limited extent. Soil sample BK has the lowest salinity and pH value but the highest values for richness and alpha diversity. However, in both soil samples B1 and B2 from the Uzen oil deposit, the salinity and pH value were the same but the richness and alpha diversity differ, with B1 being low and B2 unable to be measured. When comparing the two soil samples B1 and B3, it is noticeable that B3 had both a higher salinity and a higher pH value, but also the richness and alpha diversity were higher than in soil sample B1.

Furthermore, no clear correlation between the composition of the contaminating crude oil and richness and alpha diversity could be established, as soil samples B1 and B2 were contaminated with the same oil (Additional file 1: Table S1). The contamination level alone appeared to have a decisive influence on richness and alpha diversity in these two soil samples. Compared to these two samples, B1 and B2, the difference in richness and alpha diversity compared to B3 could be determined by the extent of contamination on the one hand, but also by the different composition of the contaminating crude oil on the other.

Richness of prokaryotes was higher in the three starting soil samples (B1, B3 and BK) compared with their corresponding enrichment culture samples (Fig. 1), suggesting the success of enrichment cultivation. Soil sample B2 was not analyzed because sufficient DNA could not be obtained, presumably due to the very high crude oil content (residual hydrocarbon contents 100,000–250,000 mg/kg soil), which hardly allows any microbial growth due to its toxicity. Although B3 was a contaminated soil sample of an oil deposit and BK was a control soil sample taken from a park close by the Almaty-1 train station, these two showed greater similarities in the microbiome of the soil sample as well as during the enrichments than comparatively the soil sample B1, also a sample of an oil deposit. These differences were also evident at the relative abundance starting at phylum level: while the relative abundance of the predominant *Actinobacteria* (B3 29.132%, BK 16.908%) and *Proteobacteria* (B3 24.386%, BK 36.781%) was relatively comparable in B3 and BK, it differed significantly in B1 with 2.629% *Actinobacteria* and 85.432% *Proteobacteria* (Additional file 1: Table S3). This was also very clear for *Acidobacteria* and *Chloroflexi*. These differences then continued at class (Additional file 1: Table S4), at order (Additional file 1: Table S5), at family (Additional file 1: Table S6) and at genus (Additional file 1: Table S7) level (Fig. 1).

#### Changes in eukaryotic microbiome composition during enrichment culture

Approximately 3.1 million raw reads were generated for eukaryotic samples. About 2.9 million high-quality reads were retained after pair-end read assembly, quality filtering and chimera removal. These processes resulted in 888 ASVs for downstream analyses.

**Fig. 2:**
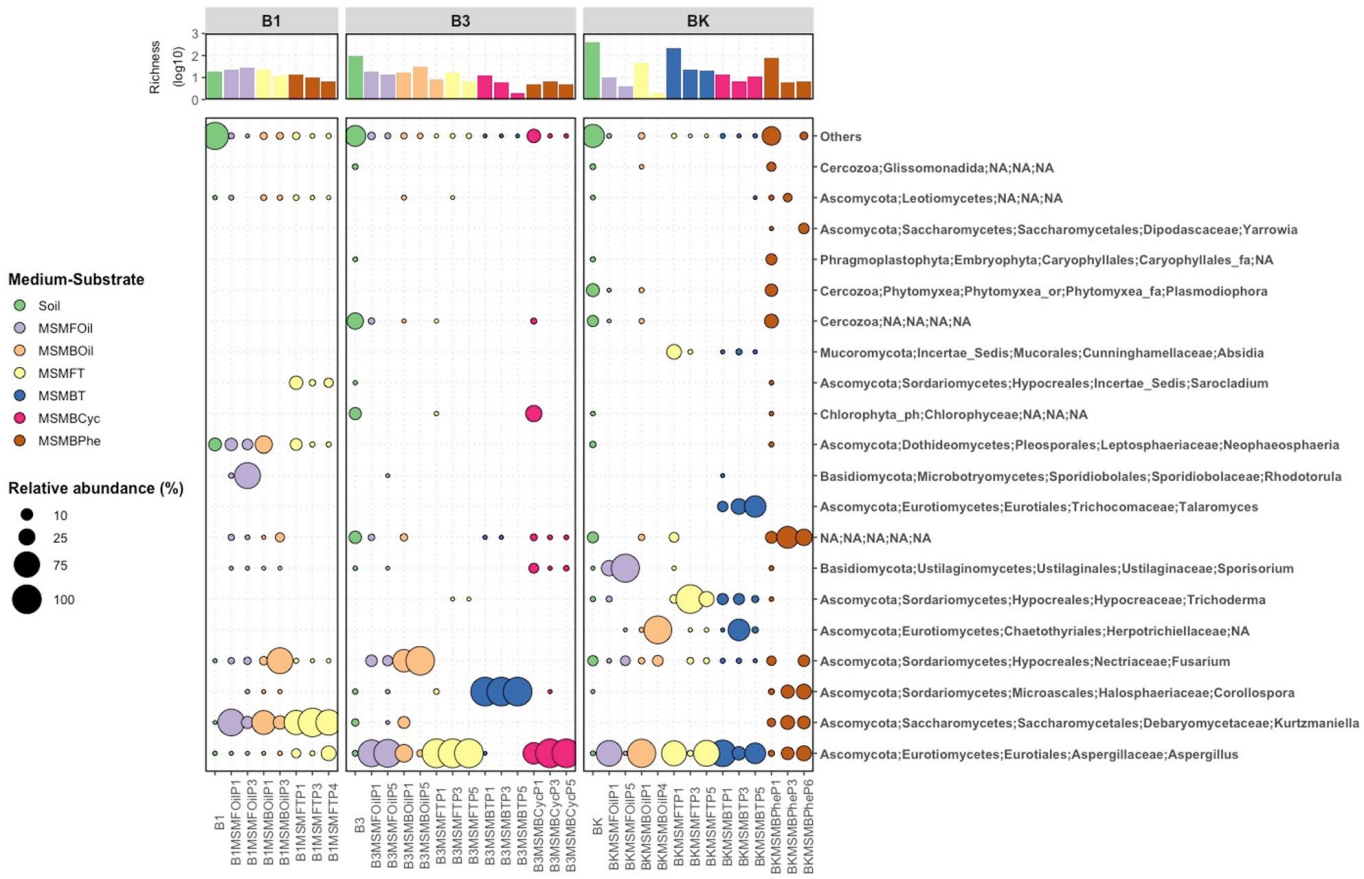
Changes in eukaryotic richness and microbiome composition during enrichment cultures. Soil: original soil sample; MSMFOil: MSMF—mineral salt medium for the isolation of yeasts and fungi, Oil—isolation substrate crude oil; MSMBOil: MSMB—mineral salt medium for the isolation of bacteria, Oil—isolation substrate crude oil; MSMFT: MSMF—mineral salt medium for the isolation of yeasts and fungi, T—isolation substrate tetradecane; MSMBT: MSMB—mineral salt medium for the isolation of bacteria, T—isolation substrate tetradecane; MSMBCyc: MSMB—mineral salt medium for the isolation of bacteria, Cyc—isolation substrate cyclohexanone; MSMBPhe: MSMB—mineral salt medium for the isolation of bacteria, Phe—isolation substrate phenol

Richness (number of ASVs) and alpha diversity of eukaryotes of the soil samples B1, B2, B3 and BK also depended from the contamination level and were therefore also not detectable in soil sample B2 with the highest residual hydrocarbon content and were highest in the control sample BK, which has the lowest value of the residual hydrocarbons (Fig. 2; Table 2).

**Table 2:** Alpha diversity of eukaryotes versus hydrocarbon contents, salinity and pH of contaminated soils in Kazakhstan

Samples	Chao 1	ASV (Richness)	Shannon	Simpson	Residual hydrocarbon contents (mg/kg soi)	Soil salinity (%)	Soil pH
B1	18	18	1.09	0.57	60,000–80,000	3–5	8.5
B2	–^a^	–	–	–	100,000–250,000	3–5	8.5
B3	96	96	3.45	0.95	10,000–30,000	7–9	9.0-9.3
BK	394	394	3.94	0.93	500–1000	0.5	5.1

^a^Soil sample was not analysed because sufficient DNA could not be obtained

A correlation between the richness and alpha diversity of eukaryotes and soil salinity and acidity could not be demonstrated. Soil sample BK had the lowest salinity and pH value but the highest values for richness, while the Shannon and Simpson values were nearly the same as for soil sample B3. However, in both soil samples B1 and B2 from the Uzen oil deposit, the salinity and pH value were the same but the richness and alpha diversity differ, with B1 being low and B2 unable to be measured. Furthermore, no clear correlation between the composition of the contaminating crude oil and richness and alpha diversity of eukaryotes could be established.

Interestingly, the richness of eukaryotes in B1 sample was much lower than in the other two samples and additionally, B3 and BK showed greater similarities in the microbiome of the eukaryotes. In soil B1, almost exclusively *Ascomycota* (40.206%) and *Basidiomycota* (59.768%) were detected for the eukaryotes, while *Cercozoa* (28.575%) and *Chlorophyta* (21.425%) were the divisions with the highest relative abundance for B3 and *Cercozoa* (29.474%) and *Nematoda* (26.989%) for BK (Fig. 2, Additional file 1: Table S3). These differences then continued at class (Additional file 1: Table S4), at order (Additional file 1: Table S5), at family (Additional file 1: Table S6) and at genus (Additional file 1: Table S7) level (Fig. 2).

#### Enrichment of genera, isolation and description of strains

In nearly all cases for both prokaryotes and eukaryotes, the enriched taxa with different media and substrates accounted for more than 50% of the total abundance in the end (Figs. 1 and 2). To investigate if species of these enriched genera are cultivatable and able to degrade or transform oil components, the last passages of enrichment cultures from the different oil-contaminated soils (B1, B2, B3, BK) were used for isolation experiments. In total, 169 different strains were isolated and cultivated from all isolation experiments with Kazakh crude oil and the model oil components tetradecane, pristane, heptamethylnonane, cyclohexanone, phenol, biphenyl and anthracene (Additional file 1: Table S2).

Of the 169 microorganisms isolated in total, 36 were isolated on tetradecane, another 32 on cyclohexanone and 19 on pristane whereas on the aromatic compounds, phenol, biphenyl and anthracene, only 12, 19 and 17 strains were isolated. This means that on the aliphatic hydrocarbons clearly more strains were found with the isolation methods used. It must be pointed out that the isolation experiments with the easily usable substrate tetradecane resulted in 36 strains, while with the multiple quaternary branched heptamethylnonane only 7 strains could be isolated.

The strains that could be further cultivated after isolation were identified and analyzed for their abilities to degrade crude oil components. Of the 169 isolated strains, 110 strains were identified by 16 S rRNA or ITS analyses (Tables 3, 4 and 5).

The most abundant eukaryotic species is *Meyerozyma guilliermondii* with 13 isolated strains and the most abundant prokaryotic genera is *Peribacillus* (11 strains) with eight strains of *Peribacillus frigoritolerans*, one strain of *Peribacillus simplex* and two strains of *Peribacillus* sp.

**Table 3:** Identified microorganisms isolated on crude oil from contaminated soils in Kazakhstan^a^

SBUG number	Genbank accession number	Best hit(s) of gene sequence analysis	Ident (%) 16 S rRNA	Ident (%) ITS	Sample site	Matched ASV	Identity (%)
Isolation substrate **Kazakh crude oil**							
*Isolation medium MSMB*							
2143 (P9K2)	PP812483	*Achromobacter insolitus*	100		BK	ASV2 (P)	100
2144 (P9K1)	PP812484	*Achromobacter insolitus*	100		BK	ASV2 (P)	100
2122 (P8K1.2)	PP812489	*Georgenia* sp.	99		B3	ASV34 (P)	100
2123 (P2K3)	PP812490	*Microbacterium* sp.	100		BK	ASV1697 (P)	100
2142 (P2K2)	PP812487	*Peribacillus frigoritolerans*	100		BK	ASV506 (P)	100
2150 (P7K3)	PP812488	*Peribacillus frigoritolerans*	100		B3	ASV506 (P)	100
2118 (P7K4)	PP812485	*Peribacillus* sp.	100		B3	ASV506 (P)	100
2128 (P8K1)	PP812486	*Peribacillus* sp.	100		B3	ASV506 (P)	100
2182 (P8K30)	PP812494	*Roseococcus* sp.	98		B3	ASV125 (P)	100
2115 (P7K2)	PP812491	*Xanthobacter* sp.	99		B3	ASV249 (P)	100
-M 1768 (P26/K2)	PP227940	*Fusarium oxysporum*		100	B3	ASV6 (E)	100
-M 1769 (P26/K1)	PP227939	*Fusarium oxysporum*		100	B3	ASV6 (E)	100
*Isolation medium MSMF*							
2149 (P12K3)	PP812492	*Amycolatopsis* sp.	100		BK	ASV52 (P)	100
2105 (P27K3)	PP812493	*Caballeronia* sp.	99		B3	ASV9 (P)	99.6
-M 1747 (P27K1)	OR335322	*Fusarium oxysporum*		100	B3	ASV6 (E)	100
-Y 2214 (V2P8K1)	PP227928	*Meyerozyma guilliermondii*		98	B1	ASV2/28 (E)	100
-Y 2219 (V2P7K1)	PP227929	*Meyerozyma guilliermondii*		99	B2	–^b^	–
-M 1770 (P27/K2)	PP227938	*Penicillium javanicum*		100	B3	ASV1/39 (E)	100

^a^Strains listed according to the alphabet separately for bacteria and fungi

^b^Soil sample was not analysed because sufficient DNA could not be obtained

**Table 4:** Identified microorganisms isolated on aliphatic compounds from contaminated soils in Kazakhstan^a^

SBUG number	Genbank accession number	Best hit(s) of gene sequence analysis	Ident (%) 16 S rRNA	Ident (%) ITS	Sample site	Matched ASV	Identity (%)
Isolation substrate **tetradecane**							
*Isolation medium MSMB*							
2121 (P39K3)	PQ814205	*Brucella intermedia*	100		BK	ASV122 (P)	99.2
2133 (P37K2)	PP831833	*Paraburkholderia graminis*	100		B3	ASV14 (P)	100
2120 (P38K2)	PP831834	*Rhodococcus qingshengii*	100		B3	ASV36 (P)	100
-Y 2223 (P39K1)	OR335328	*Exophiala* sp.		99	BK	ASV17 (E)	100
-M 1749 (P38K1)	OR335324	*Scedosporium boydii*		100	B3	ASV3 (E)	100
*Isolation medium MSMF*							
2101 (V1P5K4)	PP831832	*Achromobacter mucicolens*	100		B1	ASV2 (P)	100
2104 (P25K2)	PQ814204	*Brevibacillus* sp.	96		B3	ASV192 (P)	95.6
2148 (P17K2.3)	PP831836	*Caballeronia* sp.	99		B3	ASV9 (P)	100
2131 (P25K3.2)	PQ814200	*Neobacillus drentensis*	100		B3	ASV292 (P)	100
2132 (P25K3.1)	PQ814201	*Neobacillus drentensis*	100		B3	ASV292 (P)	100
2324 (P25K1)	-	*Psychrobacillus* sp.	97		B3	ASV925 (P)	98.4
2102 (V1P6K2)	PP831835	*Serratia plymuthica*	100		B1	ASV24 (P)	100
2113 (P34K2)	PP831837	*Xanthobacter* sp.	100		BK	ASV126 (P)	100
-M 1743(P35K1.1)	OR335321	*Aspergillus* sp.		99	BK	ASV1 (E)	100
-Y 2206 (V1P7K2)	PP227930	*Cystobasidium slooffiae*		99	B2	–^b^	–
-Y 2222 (P34K1)	OR335312	*Exophiala phaeomuriformis*		99	BK	ASV17 (E)	100
-Y 2205 (V1P5K2)	PP227931	*Meyerozyma guilliermondii*		99	B1	ASV2 (E)	100
-Y 2209 (V1P8K1)	PP227932	*Meyerozyma guilliermondii*		99	B2	–	–
-Y 2210 (V2P9K1)	OR335313	*Meyerozyma guilliermondii*		99	B2	–	–
-Y 2212 (V1P6K1)	OR335314	*Meyerozyma guilliermondii*		99	B1	ASV2 (E)	100
-Y 2218 (V1P7K1)	OR335315	*Meyerozyma guilliermondii*		99	B2	–	–
-M 1741 (P17K2)	OR335318	*Penicillium javanicum*		99	B3	ASV1/39 (E)	100
-M 1742 (P17K1)	OR335319	*Penicillium javanicum*		99	B3	ASV1/39 (E)	100
-M 1744(P17K2.1)	PP227941	*Penicillium javanicum*		99	B3	ASV1/39 (E)	100
-Y 2208 (V1P5K3)	OR335316	*Sarocladium* sp.		99	B1	ASV13 (E)	100
-M 1750 (P35K2)	OR335320	*Trichoderma harzianum*		100	BK	ASV5 (E)	100
-M 1771(P35K1.2)	PP227937	*Trichoderma harzianum*		100	BK	ASV5 (E)	100
Isolation substrate **pristane**							
*Isolation medium MSMB*							
2140 (P13K2)	PP831838	*Achromobacter spanius*	100		BK	ASV2 (P)	100
2340 (P10K1)	PQ814202	*Bordetella* sp.	100		B3	ASV1 (P)	100
2141 (P13K1)	PP831839	*Inquilinus* sp.	99		BK	ASV51 (P)	99.2
2139 (P13K3)	PP831840	*Leifsonia* sp.	100		BK	ASV58 (P)	100
2138 (P11K1)	PQ814203	*Micrococcus luteus*	100		B3	ASV1241 (P)	98.4
2107 (P13K4)	PP831841	*Rhodanobacter* sp.	99		BK	ASV31 (P)	100
2168 (P42K2.1)	PP831842	*Staphylococcus epidermidis*	100		B3	ASV1927 (P)	95.3
*Isolation medium MSMF*							
2106 (P36K3)	PP831843	*Amycolatopsis* sp.	99		BK	ASV337 (P)	100
2146 (P36K4)	PP831844	*Leifsonia* sp.	100		BK	ASV58 (P)	100
2174 (P36K4.2w)	PP831847	*Leifsonia sp.*	100		BK	ASV58 (P)	100
2145 (P36K2.2)	PP831845	*Massilia* sp.	100		BK	ASV2270 (P)	100
2103 (P36K5)	PP831846	*Paraburkholderia graminis*	100		BK	ASV14 (P)	100
-M 1751 (P36K1)	OR335323	*Purpureocillium lilacinum*		100	BK	ASV5 (E)	100
Isolation substrate **heptamethylnonane**							
*Isolation medium MSMB*							
-							
*Isolation medium MSMF*							
-Y 2215 (V2P11K3b)	OR335325	*Meyerozyma guilliermondii*		99	B1	ASV2/28 (E)	100
-Y 2213 (V2P11K3a)	OR335326	*Rhodotorula mucilaginosa*		100	B1	ASV10 (E)	100
Isolation substrate **cyclohexanone**							
*Isolation medium MSMB*							
2116 (P4K5)	PP916522	*Chryseobacterium sp.*	100		BK	ASV2204 (P)	99.6
2109 (P18K3)	PQ814208	*Georgenia* sp.	99		B3	ASV34 (P)	100
2112 (P18K4)	PQ814209	*Georgenia* sp.	99		B3	ASV34 (P)	100
2119 (P5K2)	PP916518	*Gottfriedia* sp.	100		BK	ASV1977 (P)	98.0
2108 (P18K2)	PQ814207	*Microbacterium* sp.	99		B3	ASV34 (P)	100
2117 (P3K3)	PQ814206	*Microbacterium* sp.	100		BK	ASV1697 (P)	100
2124 (P4K2)	PP916519	*Microbacterium* sp.	100		BK	ASV506 (P)	100
2169 (P40K1)	PP916523	*Neorhizobium petrolearium*	100		B3	ASV28 (P)	100
2130 (P20K1.2)	PP916520	*Peribacillus frigoritolerans*	100		B3	ASV506 (P)	100
2136 (P20K1.1)	PP916521	*Peribacillus frigoritolerans*	100		B3	ASV506 (P)	100
*Isolation medium MSMF*							
-M 1746 (V1P4K2)	PP227933	*Fusarium oxysporum*		100	B2	–	–
-M 1748 (P6K1b)	PP227942	*Fusarium oxysporum*		98	BK	ASV6 (E)	100
-Y 2207 (V1P4K1)	PP227934	*Meyerozyma guilliermondii*		98	B2	–	–
-Y 2211 (V1P4K3)	PP227935	*Rhodotorula mucilaginosa*		98	B2	–	–
-Y 2224 (P6K1a)	PP227936	*Rhodotorula mucilaginosa*		99	BK	ASV10 (E)	100

^a^Strains listed according to the alphabet separately for bacteria and fungi

^b^Soil sample was not analysed because sufficient DNA could not be obtained

**Table 5:** Identified microorganisms isolated on aromates from contaminated soils in Kazakhstan^a^

SBUG number	Genbank accession number	Best hit(s) of gene sequence analysis	Ident (%) 16 S rRNA	Ident (%) ITS	Sample site	Matched ASV	Identity (%)
Isolation substrate **phenol**							
*Isolation medium MSMB*							
2151 (P16K1.1)	PP916525	*Gordonia rubripertincta*	100		BK	ASV60 (P)	100
2147 (P16K3)	PP916524	*Pseudarthrobacter oxydans*	100		BK	ASV25 (P)	100
*Isolation medium MSMF*							
2160 (P23K1.1)	PP916527	*Peribacillus frigoritolerans*	100		B3	ASV506 (P)	100
2161 (P23K1.2)	PP916528	*Peribacillus frigoritolerans*	100		B3	ASV506 (P)	100
2162 (P23K1)	PP916529	*Peribacillus frigoritolerans*	100		B3	ASV506 (P)	100
2164 (P24K1)	PP916526	*Peribacillus simplex*	100		B3	ASV506 (P)	100
-Y 2221(V2P1K1a)	PP227943	*Meyerozyma guilliermondii*		98	B2	–^b^	–
Isolation substrate **biphenyl**							
*Isolation medium MSMB*							
2163 (P33K2)	PP919345	*Cellulosimicrobium cellulans*	99		B3	ASV95 (P)	99.6
2176 (P28K2)	PP919346	*Microbacterium* sp.	100		BK	ASV1697 (P)	100
2166 (P33K3)	PP919347	*Paenibacillus* sp.	97		B3	ASV447 (P)	96.8
2155 (P33K4)	PP919344	*Priestia aryabhattai*	100		B3	ASV893 (P)	100
2156 (P33K1)	PP919348	*Rhodococcus erythropolis*	100		B3	ASV36 (P)	100
*Isolation medium MSMF*							
2134 (P21K2.1)	PP919351	*Bordetella* sp.	99		B3	ASV1 (P)	100
2135 (P21K2.2)	PP919352	*Bordetella* sp.	99		B3	ASV1 (P)	100
2114 (P21K1)	PP919353	*Caballeronia* sp.	99		B3	ASV9 (P)	100
2175 (P28K1)	PP932500	*Leifsonia* sp.	100		BK	ASV31 (P)	100
2137 (P22K1)	PP919350	*Peribacillus frigoritolerans*	100		B3	ASV506 (P)	100
2165 (P22K2)	PP919349	*Priestia megaterium*	100		B3	ASV893 (P)	100
-Y 2216 (V2P3K1)	OR335317	*Meyerozyma guilliermondii*		99	B1	ASV2/28 (E)	100
-Y 2220 (V2P4K1)	OR335327	*Meyerozyma guilliermondii*		99	B2	–	–
Isolation substrate **anthracene**							
*Isolation medium MSMB*							
2126 (P29K2w)	PQ814210	*Arthrobacter* sp.	100		B3	ASV25 (P)	79.2
2170 (P32K1)	PP932502	*Cupriavidus alkaliphilus*	99		BK	ASV37 (P)	100
2167 (P29K1w)	PP932503	*Pseudarthrobacter oxydans*	100		B3	ASV25 (P)	100
2110 (P29K3w)	PP919354	*Pseudarthrobacter siccitolerans*	100		B3	ASV25 (P)	100
2127 (P29K2g)	PP932501	*Pseudarthrobacter siccitolerans*	100		B3	ASV25 (P)	100
2111 (P29K3g)	PP919355	*Pseudarthrobacter* sp.	100		B3	ASV25 (P)	100
2158 (P29K1g)	PP932504	*Pseudarthrobacter* sp.	99		B3	ASV25 (P)	100
2172 (P32K3)	PQ814211	*Pseudomonas putida*	100		BK	ASV104 (P)	100
2129 (P32K4)	PP932506	*Stenotrophomonas acidaminiphila*	99		BK	ASV40 (P)	94.9
2171 (P32K2)	PP932505	*Stenotrophomonas* sp.	99		BK	ASV40 (P)	94.9
*Isolation medium MSMF*							
2159 (P31K2)	PP932508	*Caballeronia* sp.	99		B3	ASV9 (P)	100
2180 (P30K2)	PP932509	*Leifsonia shinshuensis*	100		BK	ASV58 (P)	100
2154 (P31K1)	PP932507	*Priestia megaterium*	100		B3	ASV893 (P)	100
2179 (P30K1)	-	*Pseudomonas putida*	100		BK	ASV63 (P)	100
-Y 2217 (V2P12K1b)	PP227944	*Meyerozyma guilliermondii*		97	B1	ASV2/28 (E)	100

^a^Strains listed according to the alphabet separately for bacteria and fungi

^b^Soil sample was not analysed because sufficient DNA could not be obtained

All isolated strains from the soil samples B1, B3 and BK were found in the microbiome composition of the corresponding soil samples (Tables 3, 4 and 5). These isolated strains belong to different genera of the last enrichment cultures (Figs. 1 and 2; Tables 6 and 7). Some of the bacterial strains were obtained from enrichments of over 50% of the corresponding taxon (Table 6), e.g. *Achromobacter mucicolens* SBUG 2101 from 86% enrichment of *Achromobacter* on tetradecane, *Paraburkholderia graminis* SBUG 2133 from 85% not assigned *Burkholderiaceae* on tetradecane and *Caballeronia* sp. SBUG 2148 (P17K2.3) and *Caballeronia* sp. SBUG 2105 (P27K3) from 67% *Burkholderia-Caballeronia-Paraburkholderia* from two different enrichments, one on tetradecane and the other on crude oil. Others of the bacterial strains were obtained from enrichments of 10 to 50% of the corresponding taxon, e.g. *Achromobacter insolitus* SBUG 2143 and SBUG 2144 both from one 26% enrichment of *Achromobacter* on crude oil, *Georgenia* sp. SBUG 2109 and SBUG 2112 both from one 15% enrichment of *Georgenia* on cyclohexanone, *Amycolatopsis* sp. SBUG 2149 from 10% *Amycolatopsis* on crude oil, *Rhodococcus qingshengii* SBUG 2120) from 10% *Rhodococcus* on tetradecane, *Pseudarthrobacter oxydans* SBUG 2147 from 13% *Pseudarthrobacter* on phenol. All other isolates originate from enrichments in which the corresponding genus was determined to be below 10%. On the other hand, no bacterial isolates were obtained from dominantly enriched genera in some enrichment cultures. None *Achromobacter* strain could be isolated from soil sample B1 on crude oil although *Achromobacter* was enriched to 68% in mineral salt medium for the isolation of bacteria and to 72% in mineral salt medium for the isolation of fungi. Furthermore, *Parvibaculum* was enriched to 56% from soil sample B3 on crude oil in mineral salt medium for the isolation of bacteria, but no strain of *Parvibaculum* was isolated but different Bacilli, that genera were not enriched, were isolated.

In addition to the prokaryotic enrichments and isolations, the eukaryotic microbiome was also investigated, whereby the conditions were selected to enrich and isolate yeasts and filamentous fungi in particular. Some of the fungal strains were obtained from enrichments of over 70% of the corresponding taxon (Table 7), e.g. the filamentous fungi *Fusarium oxysporum* SBUG-M 1768 and SBUG-M 1769 both from 97% enrichment of *Fusarium* on crude oil and *Aspergillus* sp. SBUG-M 1743 from 78% *Aspergillus* on tetradecane and the yeasts *Meyerozyma guilliermondii* SBUG-Y 2205 and SBUG-Y 2212 (Synonym *Candida guilliermondii*) both from 74% *Kurtzmaniella-Candida*-clade on tetradecane. However, strains of fungi were also isolated from enrichments of 10 to 50% of the corresponding taxon, e.g. *Meyerozyma guilliermondii* SBUG-Y 2214 (Synonym *Candida guilliermondii*) from 11% *Kurtzmaniella-Candida*-clade on crude oil and *Trichoderma harzianum* SBUG-M 1750 and SBUG-M 1771 both from 20% *Trichoderma* on tetradecane. Similar to prokaryotes, fungal strains of less enriched or undetectable taxa were also isolated and no fungal isolates were obtained from dominantly enriched genera in some enrichment cultures.

**Table 6:**
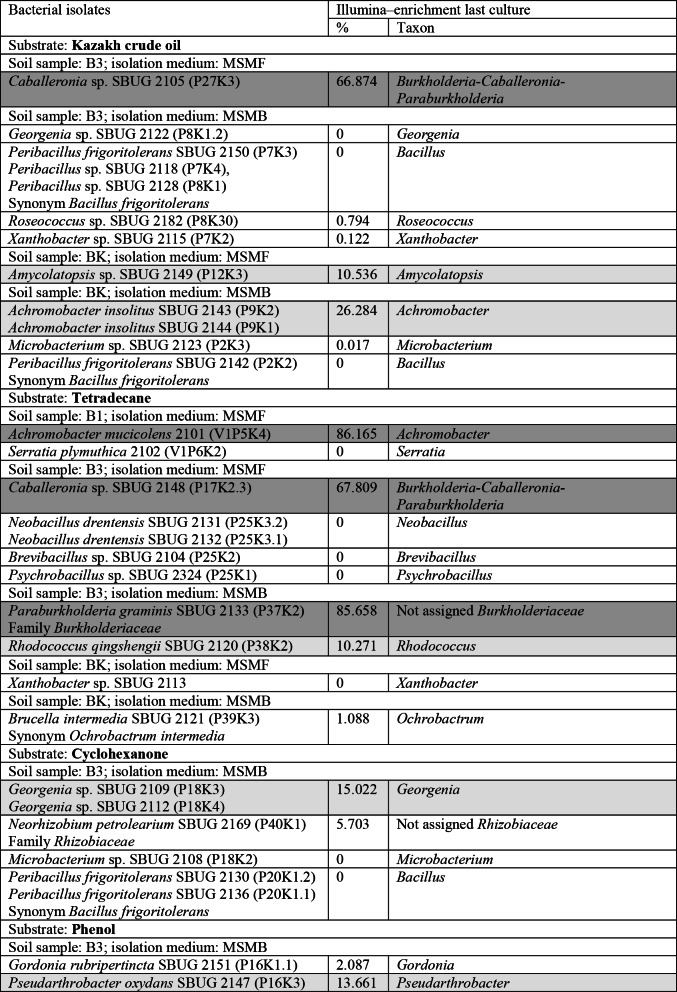
Identified bacteria isolated from contaminated soils in Kazakhstan on different substrates compared with the relative abundance of bacterial genera at the end of enrichment by illumina

Lines highlighted in dark gray indicate an enrichment of > 50%

Lines highlighted in light gray indicate an enrichment of 10–50%

**Table 7:**
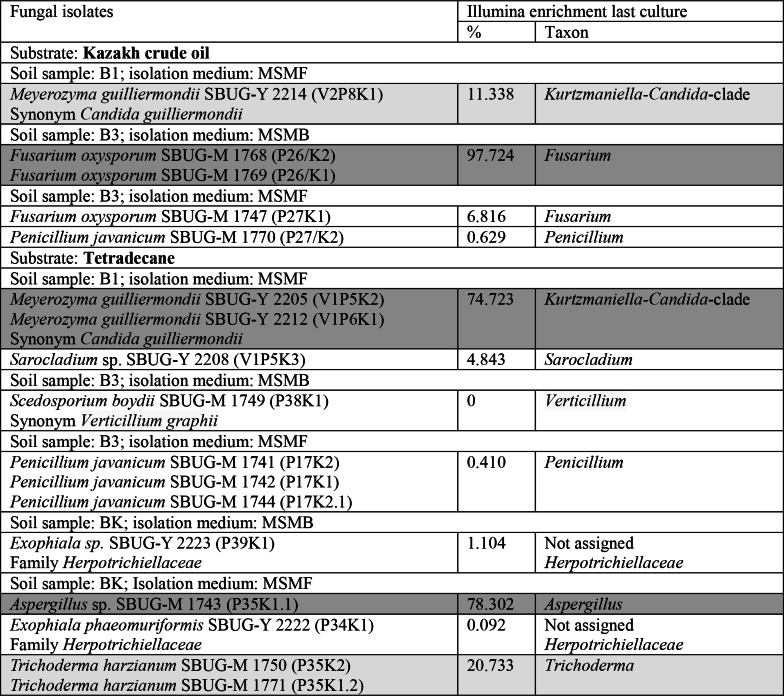
Identified fungi isolated from contaminated soils in Kazakhstan on different substrates compared with the relative abundance of fungal genera at the end of enrichment by illumina

Lines highlighted in dark gray indicate an enrichment of > 50%

Lines highlighted in light gray indicate an enrichment of 10 to 50%

#### Degradation potential of the isolated strains

To investigate the degradation and/or transformation potential of the isolated microorganisms the strains were incubated with one of the isolation substrates. Most of the strains were cultivated on the original isolation substrate with exception of the crude oil isolates which were mostly tested on the more easily useable aliphatic hydrocarbon tetradecane. Transformation experiments of all strains were conducted in liquid medium, and the metabolites were detected by GC-MS or HPLC (Additional file 1: Tables S8–S13).

26 of 40 on tetradecane tested strains were able to transform 20% or more of this substrate, 18 of them more than 70%, and 12 of these strains more than 90% of it (Additional file 1: Table S7). Two or more monocarboxylic acids, mostly myristic (1-tetradecanoic) and lauric (1-dodecanoic) acid, were detected as transformation products. The detection of monocarboxylic acids provides evidence for monoterminal degradation pathway of tetradecane. Furthermore, a dicarboxylic acid hexanedioic acid was analyzed for seven strains. But only two different species, *Meyerozyma guilliermondii* (SBUG-Y 2205, 2209, 2210, 2212, 2218, 2219) and *Scedosporium boydii* (SBUG-M 1749), could be found as dicarboxylic acid producer. The formation of dicarboxylic acids indicates a diterminal degradation pathway of tetradecane for these two species. Furthermore, 11 strains formed ketones during the transformation of tetradecane. The detection of ketones indicates subterminal degradation of *n*-alkanes, in our studies of tetradecane. Eight of these strains produced these ketones in addition to the monocarboxylic acid(s), suggesting that a combination of mono- and subterminal degradation could also be assumed for these strains. But the GC-MS analyses of the culture medium of two of these strains showed only the production of ketones coupled with a degradation rate of more than 70% of the substrate, which corresponds to a very good degradation rate for tetradecane, but does not allow any further conclusions to be drawn about the further degradation pathway starting from the ketones. In summary, the strains isolated on tetradecane can form mono- and dicarboxylic acids and ketones, thereby utilizing *n*-alkanes via the mono-, di- and subterminal degradation pathway.

Only four of the 14 on pristane tested strains were even able to accomplish a decrease of this substrate during the incubation (Additional file 1: Table S8). Whereas the two bacteria *Leifsonia sp.* SBUG 2174 and *Staphylococcus epidermidis* SBUG 2168 could only use 55.6 or 10.0% of the substrate, the two filamentous fungi *Purpureocillium lilacinum* SBUG-M 1751 and *Fusarium oxysporum* SBUG-M 1747 transformed 99.5 or 95.8% of the substrate pristane. The GC-MS analyses of all four pristane degraders showed the production of branched chain dicarboxylic acids, such as 2,6-dimethylheptanedioic acid, 2-methylpent-2-enedioic acid, 2-methylpentanedioic acid and 2-methylbutanedioic acid indicating a diterminal degradation pathway for pristane.

None of the two on heptamethylnonane isolated strains *Meyerozyma guilliermondii* SBUG-Y 2215 and *Rhodotorula mucilaginosa* SBUG-Y 2213 were able to degrade or transform this substrate under the given cultivation conditions during the given incubation time. However, the species *Meyerozyma guilliermondii* was also isolated on all other substrates excepted pristane and the species *Rhodotorula mucilaginosa* was also found on cyclohexanone.

Various isolates (16) were tested to transform cyclohexanone (Additional file 1: Table S10). Some of them (11) were cyclohexanone degraders in which nine were able to metabolize more than 60% of this substrate. Metabolites were detected for all 16 isolates. ε-Caprolactone and hexanedioic acid as intermediates indicate an almost complete degradation of cyclohexanone via ring cleavage and further degradation by ß-oxidation, whereas the sole detection of cyclohexanols would indicate only a transformation of the substrate.

More than 40% of the on phenol tested strains were able to transform this substrate nearly completely whereas the other strains showed no decrease of the substrate although different metabolites could be detected for two of these no-degraders (Additional file 1: Table S11). Since, except for one strain *Pseudarthrobacter oxydans* SBUG 2147, mainly quinones and/or dimers were detected, we cannot speak of phenol degraders but at most of transformers. The detection of muconic acid as degradation product of *Pseudarthrobacter oxydans* SBUG 2147 suggests *ortho*-ring cleavage of the aromatic ring of the phenol.

Of the 13 strains isolated and cultivated on biphenyl, monohydroxylated products were detected in the culture supernatants of seven strains and dihydroxylated products in the supernatant of three strains (Additional file 1: Table S11). In addition, unknown products were measured by HPLC and GC-MS in the supernatant of six strains. Ring cleavage products such as 4-phenyl-2-pyron-6-carboxylic acid and (5-oxo-3-phenyl-2,5-dihydrofuran-2-yl)acetic acid could only be clearly identified in the case of the two strains of *Priestia aryabhattai* SBUG 2155 and *Priestia megaterium* SBUG 2165. The formation of these metabolites suggests *ortho*-ring fission of one of the two aromatic rings of biphenyl. Due to the very poor solubility of biphenyl in aqueous solutions, the decrease of this substrate was not determined in the culture supernatants.

Also, in the case of anthracene, the substrate decrease in the culture supernatants was not determined due to the very poor solubility in aqueous solutions. Of the 15 strains isolated and cultivated on anthracene, transformation products were detected in the culture supernatants of 11 strains, among which mainly unknown metabolites or simple oxidation products like anthrone and 9,10-anthracendione were detected (Additional file 1: Table S13). There were only indications of possible degradation products, 3-hydroxy-2-naphthoic acid or muonic acid, for the two strains *Stenotrophomonas* sp. SBUG 2171 and *Pseudarthrobacter oxydans* SBUG 2167. These two products also indicate *ortho*-ring cleavage of the aromatic rings of anthracene.

In summary, aliphatic substrates such as tetradecane and pristane can be transformed by the isolated strains via various degradation pathways (mono-, di- and subterminal degradation), whereas the ring cleavage of aromatic substrates such as phenol, biphenyl, and anthracene after initial hydroxylation presumably only proceeds via *ortho*-ring fission, based on the detected cleavage products.

## Discussion

### Definition of oil-contaminated soils

About 1000 mg crude oil per 1 kg soil has already been described as oil contamination by other authors [34, 45], approximately 1000 mg hydrocarbons per 1 kg soil as hydrocarbon contamination [46], 2.34% total petroleum hydrocarbons of soil weight (means 23,400 mg per 1 kg soil) as lightly polluted soil [42], 4000–20,000 mg per kg as medium and over 20,000 mg per kg soil as heavy petroleum concentrations [40]. But on the other hand, an oil contamination of 6800 mg/kg soil was used as a control soil [42]. All these designations and their values show very clearly that great care must be taken when interpreting and comparing data from different experimental settings. For this reason, we have also dispensed with the designation as heavily, lightly or non-contaminated soil and continued to use our BK soil sample as a control sample, because of the lowest value of residual hydrocarbon (500–1000 mg/kg soil) and with 1219 ASVs as the highest value of richness of our samples. Nevertheless, B3 has the lowest content of residual hydrocarbons (10,000–30,000 mg/kg soil) and the highest richness (425 ASVs) of all our oil-contaminated samples, followed by B1 (residual hydrocarbons 60,000–80,000 mg/kg soil, richness 160 ASVs). B2 had the highest content of residual hydrocarbons (100,000–250,000 mg/kg soil) and it was not possible to isolate a sufficient amount of DNA, and therefore no richness could be determined.

### Microbial richness and diversity

Richness (number of ASVs) of prokaryotes (Fig. 1; Table 1) and eukaryotes (Fig. 2; Table 2) and alpha diversity (Chao1, Shannon and Simpson, Tables 1 and 2) of the soil samples decreased with increasing contamination level and was therefore highest in the control sample BK, which has the lowest value of the residual hydrocarbons, followed by the oil-contaminated samples B3, B1 and B2. This suggests that oil contamination has a negative impact on microbial diversities as only certain microbes could tolerate the toxicity of crude oil. Other studies also found that there exists a large difference in the alpha diversity of bacteria in uncontaminated and oil-/ hydrocarbon-contaminated soil samples [32, 40, 45, 46], whereby the differences are partly comparable to those of our samples BK and B3. Thus, our low or undeterminable values of the richness of our soils B1 and B2 also fit into this line, that the diversity decreased with increasing contamination levels as described by Geng at al. [64]. However, there are also studies in which the degree of oil contamination is similar to ours from soil B3, but the alpha diversity (Chao1) is five to ten times higher [38], which could presumably be due to the different composition of the various crude oils and the different nature of the soils. A few additional studies also showed that oil contaminations reduced the bacterial diversity of soils in comparison with control samples [34, 40, 42]. A similar trend, that oil contamination decreased bacterial overall diversity, were observed in field experiments with artificially added motor oil [36] or kerosene [39] in the first year of the studies, but in the following years this dependency was no longer detectable and the bacterial communities changed over time, regardless of oil addition and the differences over time were more significant than the differences between oil contaminated and non-contaminated samples [36].

Furthermore, strong differences in eukaryotic diversity comparable to our results (Fig. 2; Table 2) could be found in hydrocarbon-contaminated and uncontaminated soil samples [42, 46] and additionally, the diversity decreased with increasing contamination levels Geng at al. [64]. However, there are also studies in which the differences in fungal diversity between contaminated and uncontaminated samples are not quite so clear as in bacterial communities of the same soil samples [45]. In addition to contaminated soil samples with a lower Chao index of fungal diversity, equivalently contaminated soil samples with a higher Chao index of fungal diversity comparable to that of the control sample were also detected [45].

### Prokaryotic microbial communities

Our different soil samples showed a varying response in terms of microbial taxa to the different levels of hydrocarbon contaminations (Fig. 1, Additional file 1: Tables S3–S7). At the phylum level soil B1 was predominated by *Proteobacteria* with 85% and soil BK with 37%, whereas B3 was dominated by *Actinobacteria* with 29% followed by *Proteobacteria* with 24%. The second most represented phylum of BK was *Acidobacteria* with 21% and of B1 *Firmicutes* with 10%. *Proteobacteria* was mostly described as the predominant phylum in oil-polluted soils with varying values: 77% [44], around 75% [32], 20–77% [35], around 45–55% [45], 46–54% [38], 44% [37], around 40% for soil taken directly from oil wells [33], around 35–85% for heavy petroleum-contaminated soils [40] and in different contaminated soil samples: with around 40% in fields that were used in the past for illegal waste disposal and dumping [46], and with 77% in polluted soil of a polyether glycol factory field with mainly petroleum hydrocarbons as pollutants [44]. The dependence of the prokaryotic taxa on the degree of contamination becomes particularly clear in Geng et al. [64] and Shelyakin et al. [39], where heavily polluted soil samples showed 55–84% *Proteobacteria*, while lightly polluted ones had only 6–30% *Proteobacteria* with the predominant phylum *Actinobacteria* with 27–42%, which are almost in the range with our results if we were to classify B1 as heavily contaminated and B3 and BK as lighter contaminated.

On genus level soil B1 was predominated by *Pseudomonas* (82%) of phylum *Proteobacteria* followed by *Carnobacterium* (8%) of phylum *Firmicutes*, while B3 and BK are much more diverse. *Mycobacterium* (6%) and *Nocardioides* (4%) of phylum *Actinobacteria*, *Caloribacterium* (3%) of phylum *Firmicutes* and *Acidithiobacillaceae* (KCM-B-112 with 14%) of phylum *Proteobacteria* are the most abundant genera/family in soil B3 alongside many prokaryotes unclassified at genus or higher taxa level and over 40% other genera that do not exceed the 3% relative abundance. *Pyrinomonadaceae* (RB41 with 7%) of phylum *Acidobacteria* and *Methylotenera* (13%) of phylum *Proteobacteria* are the most present genera/family in soil BK, but over 60% of prokaryotes do not exceed the 3% relative abundance or are unclassified at genus or higher taxa level, which clearly shows the significantly higher prokaryotic diversity of BK compared to B1.

The genus-level analyses of other bacterial compositions in crude oil contaminated soils also led to many unclassified results [33] up to almost 58% in some cases [36] and the species-level analyses produced poor results in principle, as many of the sequenced species are uncultivated and therefore unclassified [33]. Overall, the data from other authors on the prokaryotic response on genus level in oil contaminated soils showed great diversity as our data do. For two chronic refined petroleum oil contaminated soils from India the genera *Gordonia* (13%) of phylum *Actinobacteria* was dominant for one soil and *Nesterenkonia* (17%) of phylum *Actinobacteria* of the second soil, for another two persistent crude oil contaminated soils for one *Staphylococcus* (44%) of phylum *Firmicutes* and for the second *Chryseobacterium* (10%) and *Mycobacteria* (7%) of the phyla *Bacteroidetes* and *Actinobacteria*, whereas *Pseudomonas* was found in every soil but dominate only in one with 9% [38]. *Pseudomonas* (6.6%) were analysed for polluted soil samples of a polyether glycol factory in China, whereas *Acinetobacter* (29%, phylum *Proteobacteria*), an unspecified genus (PYR10d3 with 19.5%) and *Dietzia* (11.4%, phylum *Actinobacteria*) were more dominant on the genus level [44]. For seven oil and gas drilling sites in India the phylum *Proteobacteria* (52–70%) was also the most prevalent followed by *Actinobacteria*, *Acidobacteria*, *Firmicutes*, *Bacteroidetes* and on genus level *Mycobacterium* (7.9%), *Burkholderia* (5.5%), *Pseudomonas* (3.1%), *Bacteroides* (2%), *Bradyrhizobium* (3.2%), *Acidovorax* (2.9%), *Aromatoleum* (1.4%), *Methylobacterium* (1.2%), and *Phenylobacterium* (1.1%) [31] and was therefore much more diverse than our soil B1. In artificial prepared oil contaminated soils, the phyla *Proteobacteria* and *Bacteroidetes* were significantly abundant with *Burkholderia*, *Dechloromonas*, *Erythrobacter*, *Hydrocarboniphaga*, *Hyphomicrobium*, *Lamia*, and *Methyloversatilis* on genus level [34]. Families of *Alphaproteobacteria* like *Acetobacteraceae*, *Bradyrhizobiaceae*, *Hyphomicrobiaceae*, *Rhizobiaceae*, *Rhodobacteraceae*, and *Sphingomonadaceae* were dominant in soils taken directly from oil wells in Poland [33] and are quite different to our results on family level (Additional file 1: Table S6). All these examples together with our results and a very comprehensive compilation of bacterial communities from 12 different oil-contaminated habitats including arctic, taiga, and marine sediments [35] impressively demonstrate that the prokaryotic taxonomic composition of all these habitats varied significantly.

### Eukaryotic microbial communities

The composition of eukaryotic communities of heavy hydrocarbon polluted soils was strongly dominated by *Ascomycota* (70–87%) with 50–70% *Sordariomycetes* in class level [46], whereas in our studies 60% of the eukaryotic community were *Basidiomycota* and only 40% *Ascomycota* for soil B1 (Additional file 1: Table S3) and in class level mainly *Microbotryomycetes* (59%) followed by *Dothideomycetes* (40%) (Additional file 1: Table S4). Our soil B3 were dominated by *Cercozoa* (29%) and *Chlorophyta* (21%) followed by *Ochrophyta* (12%), whereas *Ascomycota* with 5% and *Basidiomycota* with 1% only played a subordinate role (Additional file 1: Table S3). Again, *Cercozoa* with 29% dominated soil BK together with *Nematoda* with 27% followed by *Ascomycota* (12%), *Ciliophora* (9%) and *Ochrophyta* (7%). *Cercozoa*, *Chlorophyta*,* Ciliophora*,* Nematoda* and *Ochrophyta* play no or a very subordinate role in heavily and lightly petroleum hydrocarbon contaminated soils from an oilfield in China [64]. The average abundance of *Ascomycota* in these heavily petroleum hydrocarbon contaminated soils was found to be 40% and therefore lower than the average abundance in the lightly contaminated soils with 65%. The value for the *Ascomycota* in these highly contaminated soils corresponds very well with that for our B1, which also has a high contamination, but the values for our less contaminated soils show very different patterns.

*Ascomycota* and *Chytridiomycota* were the main fungal division in PAH contaminated coastal marine sediments [43] and *Ascomycota* (40–80%) and *Basidiomycota* (20–60%) in oil polluted subarctic soils [45] and also *Ascomycota* in heavily oil polluted soil [42]. *Alternaria* (40.8%) were analysed for polluted soil samples of a polyether glycol factory in China, and *Fusarium* (22.5%) and *Aspergillus* (11.4%) followed, *Ascomycota* in total 97.6% [44]. On genus level our soil B1 was dominated by an unclassified genus of the family *Sporidiobolaceae* with 59% (division *Basidiomycota*), but followed by the *Ascomycota Alternaria* (24%) and *Neophaeosphaeria* (13%). In our soil BK, *Fusarium* was detected at 7% and *Neophaeosphaeria* at 1%, and thus played a subordinate role, just like in soil B3, in which they were not detectable at all. Thus, the data on eukaryotes also show that the taxonomic composition of the contaminated habitats varied significantly. However, little data are available for eukaryotes, further research would be necessary to obtain a comprehensive overview as for the prokaryotes.

### Enrichment and isolation–comparison of culture-dependent and -independent results

Furthermore, in our studies a comparison was performed between the isolated strains and the culture-independent enrichment of microorganisms. Only Spini et al. [2] presented a comparison between culture-dependent and culture-independent analyses comparable to ours. While Spini et al. [2] found that the relative Illumina abundance and the isolation values matched for most of the samples, our results are much more diverse. Some bacterial strains as *Achromobacter mucicolens* 2101, *Paraburkholderia graminis* SBUG 2133 and *Caballeronia* sp. SBUG 2105 and SBUG 2148 were isolated from enrichment cultures, that were 67–86% enriched by the corresponding taxon (Table 6), while mainly *Pseudomonas* was enriched and isolated by Spini et al. [2]. Other of our bacterial strains originated from enrichments of 10–50% of the corresponding genera. Comparable results were obtained for fungi. The filamentous fungi *Fusarium oxysporum* SBUG-M 1768 and SBUG-M 1769 were isolated from enrichment cultures of 97% *Fusarium*. Spini et al. [2] also mainly enriched and isolated *Fusarium* strains (40–69%). *Aspergillus* sp. SBUG-M 1743 and the yeasts *Meyerozyma guilliermondii* SBUG-Y 2205 and SBUG-Y 2212 were isolated from enrichment cultures, that were 70–80% enriched by the corresponding genera (Table 7). Other isolates were obtained from enrichments of 10–50%. However, there were also isolates, that originate from enrichments in which the corresponding genus was determined to be below 10%.

It is noteworthy that strains were isolated from some enrichments in which 0% of the corresponding genus was detected, which particularly concerns the bacterial genus *Bacillus* in our studies. Spini et al. [2] also described isolates from enrichments in which no sequences affiliated to the corresponding genera. On the other hand, no isolates were obtained from dominantly enriched genera in some of our enrichment cultures and also in the analyses of Spini et al. [2]. However, it is well known that microbial specialists that are particularly good degraders, but cannot be isolated and cultivated under laboratory conditions, survive and accumulate in acutely contaminated soils. The problem of non-cultivable microorganisms and the associated underestimation of the existing microbiological community is a recurring theme [2, 65]. There are particular difficulties in assessing the microbiological community of contaminated soils in connection with isolated individual organisms.

**Fig. 3:**
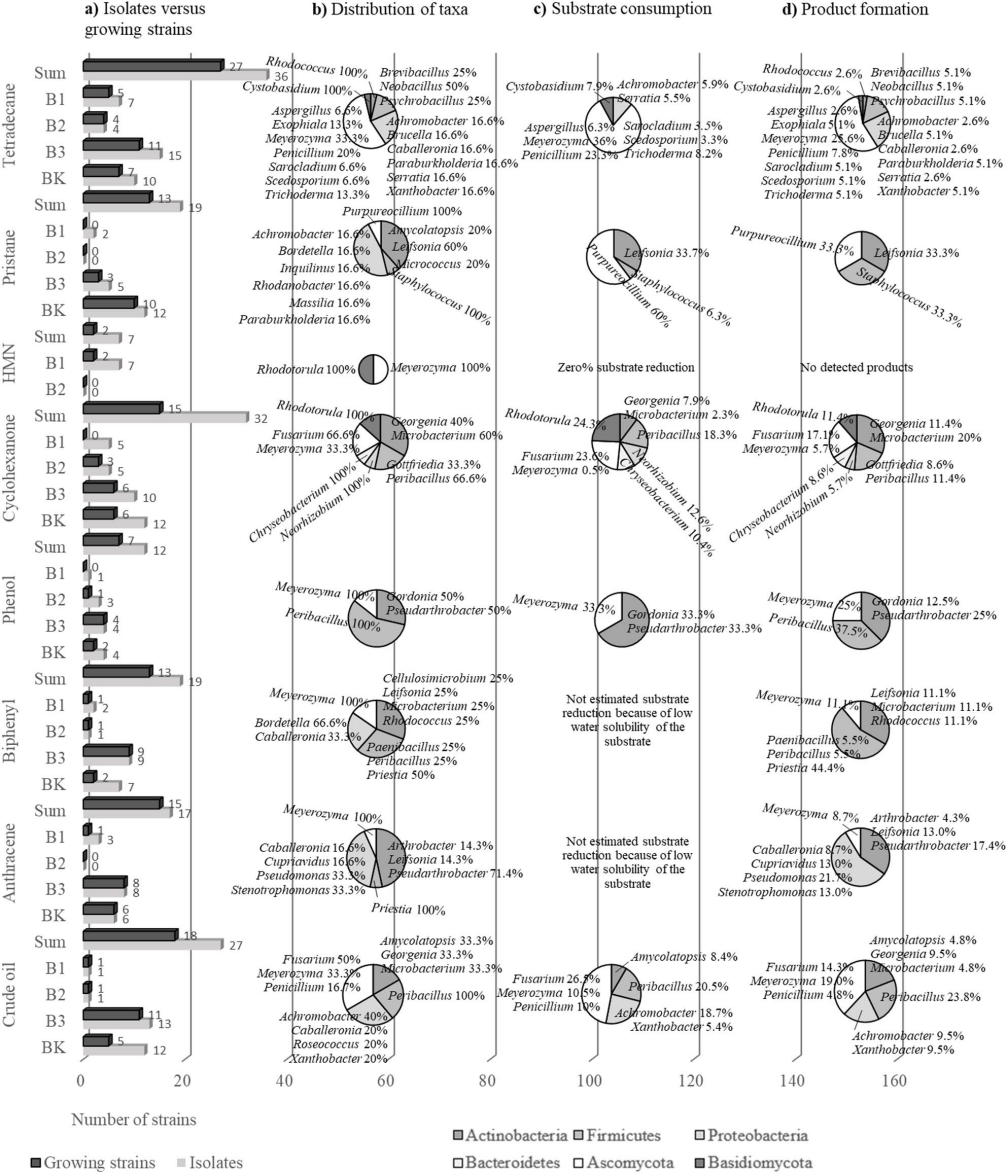
Overview of the isolation substrates (left side), the isolated strains (numbers and diversity) and their degradation capabilities (The circular charts in horizontal orientation each belong to the substrate named on the left side). **a** Number of isolated microorganisms and number of taxonomical characterized strains per isolation substrate and soil sample, and the sum of strains per substrate, **b** Percentage distribution of phyla/divisions and genera per substrate, diversity of genera within the phyla/divisions is illustrated by genus names and percent values, **c** Percentage distribution of phyla/divisions and genera regarding the substrate consumption per substrate, **d** Percentage distribution of phyla/divisions and genera regarding the product formation per substrate

In this work, we combined the microbiome insights on the enrichment procedures for the isolation of crude oil degrading bacteria, yeasts and filamentous fungi with the in-fact degradation potential of the isolated species. Of the 169 strains isolated, a total of 94 were obtained from the aliphatic compounds tetradecane (36), pristane (19), heptamethylnonane (7) and cyclohexanone (32), while only 48 strains grew on the aromatic structures phenol (12), biphenyl (19) and anthracene (17) (Fig. 3A, Additional file 1: Table S2). Additional 27 strains were isolated from crude oil itself. Comparable results were obtained from Spini et al. [2], whereby more strains on aromatic substrates were isolated.

### Degradation of oil components by isolated microbial strains

Of the 169 strains isolated in total, 110 strains could be taxonomically determined after isolation and tested for their ability to transform oil components. For all substrates, strains were isolated that could not be cultivated further, but their number varied from substrate to substrate (Fig. 3a). In total, we described 110 oil component degraders belonging to the bacterial phyla *Actinobacteria*, *Bacteroidetes*, *Firmicutes* and *Proteobacteria* and to the fungal divisions *Ascomycota* and *Basidiomycota*. The occurrence and percentage distribution of the phyla/divisions varied greatly between the different substrates (Fig. 3b). Only cyclohexanone converting strains could be described from the six phyla/divisions mentioned and only the fungal division *Ascomycota* could be found for all substrates. *Actinobacteria*, *Firmicutes* and *Proteobacteria* were present for almost all substrates. In addition, the genera within the individual phyla also varied from substrate to substrate in terms of quality and percentage quantity, e.g. for the fungal division *Ascomycota*, seven different genera with a percentage share of 6.6% (*Aspergillus*, *Sarocladium*, *Scedosporium*), 13.3% (*Exophiala*, *Trichoderma*), 20% (*Penicillium*) and 33% (*Meyerozyma*) within the *Ascomycota* were described for the substrate tetradecane, while for the substrates phenol, biphenyl and anthracene only the genus *Meyerozyma* was isolable and cultivable for *Ascomycota*.

The greatest diversity of isolated genera was detected on the substrate tetradecane with 18 different genera, followed by pristane with 11, biphenyl with 10, cyclohexanone and anthracene with 9 each, and heptamethylnonane with only 2 genera. It can be deduced, strains that degrade simple aliphatic substrates such as tetradecane can be isolated more easily than strains that degrade branched chain substrates such as pristane and heptamethylnonane. As the degree of branching points increases, the isolation of strains decreases. The two strains isolated on heptamethylnonane were then unable to reduce the substrate or form products. The diversity of strains that can be isolated on alicyclic substrates such as cyclohexanone was also less than that of tetradecane. The isolation of different genera on aromatics was also more difficult than that on tetradecane as isolation substrate.

But we not only showed the presence and the possibility of enrichment and isolation of strains on these compounds but also the degradation potential of the isolated bacteria, yeasts and filamentous fungi based on substrate consumption (Fig. 3c) and product formation (Fig. 3d, Additional file 1: Tables S8–S13). In principle, fewer strains are able to reduce the substrate quantity than strains can be isolated and cultivated, which could be determined for all substrates. Furthermore, the different phyla/divisions and within the phyla/divisions also the different genera are involved in the degradation to different degrees (Fig. 3c). Another parameter for describing substance conversions is the formation of products from the corresponding substrates. In contrast to substrate reduction, strains from most of the phyla and genera described are involved in product formation from the substrates used. Exceptions are the substrates pristane, for which only 3 of the 13 strains analyzed showed substrate reduction and product formation, and heptamethylnonane, for which neither substrate reduction nor product formation was observed.

The most abundant species is the ascomycetal yeast *Meyerozyma guilliermondii* and therefore the most abundant genera is *Meyerozyma* (Fig. 3, Additional file 1: Tables S8–S13). *Meyerozyma guilliermondii* were isolated on almost all substrates, exception pristane, and is responsible for substrate reduction and product formation also for almost all substrates, exception pristane and heptamethylnonane. Compared to all strains isolated on tetradecane, *Meyerozyma guilliermondii* contributes 36% to substrate reduction and 25.6% to product formation (Fig. 3c and d). For the substrate phenol, the proportion of total degradation and product formation of *Meyerozyma guilliermondii* is comparable to the rates for tetradecane degradation, but lower for the substrates biphenyl and anthracene. These results are not surprising, as *Meyerozyma guilliermondii* (synonym *Pichia guilliermondii* and asexual or anamorphic form known as *Candida guilliermondii*) has been repeatedly isolated from various contaminated habitats for decades and has been described as a good utilizer of aliphatic and aromatic compounds [28, 66–71] and more recently even as a polyethylene degrader [72, 73].

Within the described prokaryotes, the genus *Peribacillus* is the most abundant, although it could only be isolated and cultivated on the four substrates crude oil, cyclohexanone, phenol and biphenyl (Fig. 3a and b). Only for cyclohexanone a substrate reduction but for the other substrates also a product formation could be detected for the genus *Peribacillus*. In this context, it is surprising that no *Peribacillus* strain could be isolated on tetradecane, since all *Peribacillus* strains isolated from crude oil can reduce and metabolize tetradecane (Fig. 3, Additional file 1: Table S8). *Peribacillus frigoritolerans* is the most abundant species of the genus *Peribacillus* in our studies. There is nothing described about the degradation potential of *Peribacillus frigoritolerans* concerning aliphatic and aromatic oil components, but there are reclassifications of *Brevibacterium frigoritolerans* to *Peribacillus frigoritolerans* and also to *Bacillus frigoritolerans* [74, 75]. Thus, *Bacillus frigoritolerans* is named as a species in a consortium that consists of different *Bacillus* strains and is capable of degrading pyrene, a condensed aromatic compound [76]. *Brevibacterium frigoritolerans* is described as organism for the bioremediation of phorate, an organophosphorus insecticide [77–80] and the degradation of paracetamol, an acetaminophenol [81]. *Peribacillus frigoritolerans* is described to be able to grow on different polymers [82, 83]. Apart from this, however, the importance of the genus *Bacillus* in the conversion of hydrocarbons and environmental pollutants is widely known [23, 84–87].

While strains from the named genera have been described extensively as hydrocarbon degraders, little has been described for different species from the fungal genera *Sarocladium*, *Purpureocillium* and *Rhodotorula.* One strain of *Sarocladium*, a *Sarocladium* sp. SBUG-Y 2208, was isolated on tetradecane and was able to reduce the substrate concentration and to form metabolites (Fig. 3, Additional file 1: Table S8). The genus *Sarocladium* include around 20 species belonging to the division *Ascomycota*. Many of these species have been transferred from the genus *Acremonium* to *Sarocladium* [88, 89]. While *Sarocladium* species are little known as alkane degraders [55], several *Acremonium* species have been described as hydrocarbon-assimilating [90–93].

*Purpureocillium lilacinus* SBUG-M 1751 was isolated on pristane and was shown in this work as powerful degrader of this substrate. *Purpureocillium lilacinus* was previously known as *Paecilomyces lilacinus* [94]. *Paecilomyces lilacinus* was described as degrader of aromatic compounds such as toluene [95], biphenyl and dibenzofuran [96] or benzo(a)pyrene [97] for many years. Only recently, the utilization of aliphatic compounds such as *n*-alkanes, branched chain alkanes and cycloalkenes was also reported [55, 98–100].

Two strains of *Rhodotorula mucilaginosa* (SBUG-Y 2211, SBUG-Y 2224) were isolated on cyclohexanone and one (SBUG-Y 2213) on heptamethylnonane (Fig. 3b). While almost 100% of cyclohexanone was converted via the previously described microbial pathway to ε-caprolactone and hexanedioic acid [101], heptamethylnonane could not be utilized. In this context, it should also be mentioned that heptamethylnonane is a branched chain aliphatic hydrocarbon with 3 quaternary and one tertiary carbon atom and the isolation of microorganisms on this substrate is astonishing in itself. *Rhodotorula mucilaginosa* has been little described as degrader of aromatics [71, 102–104] and more recently of cyclohexanone and *n*-alkanes [55], however, the use of alkanes is controversial [105]. The species *Rhodotorula mucilaginosa* has so far received little attention in the field of aliphatic and alicyclic hydrocarbon degradation, but overall were now isolated from five different soil samples in Kazakhstan (B1, B2 and BK in this investigation and E3 and E4 in Mikolasch et al. [55]). The greatest distance between the soil extraction points is around 2000 km / 1243 miles indicating that this species is widespread in contaminated soils and can be considered to have a high potential for degradation.

Furthermore, little is known about the degradation of different hydrocarbons for some bacterial species or genera examined in this study. *Neorhizobium petrolearium* SBUG 2169 was isolated on cyclohexanone and is an excellent utilizer of this substrate (100%) with the formation of transformation products (Fig. 3c and d, Additional file 1: Table S10). To date, there is no report concerning cycloalkane utilization by *Neorhizobium petrolearium*. This species was recently reclassified from the species *Rhizobium petrolearium* [106]. *Rhizobium petrolearium* was first described in 2012 as a bacterium isolated from oil-contaminated soil [107] just like our strain SBUG 2169, suggesting that such strains can utilize petroleum components well. The utilization of PAHs such as anthracene, fluorene, naphthalene, phenanthrene and pyrene was mainly investigated for *Rhizobium petrolearium* [107–109]. Very recently the degradation of crude oil and its derivative fuels was mentioned for *Rhizobium petrolearium*, although these studies mainly focused on the utilization of *n*-alkanes and branched chain alkanes [110]. Together with our studies on the degradation of cyclohexanone by *Neorhizobium petrolearium* SBUG 2169, *Rhizobium/Neorhizobium petrolearium* can now be described as a utilizer of aliphatic, alicyclic, aromatic and PAHs, should therefore be able to convert a large number of crude oil components and can thus be attributed a very high potential in bioremediation.

A total of 5 strains of the genus *Leifsonia* were isolated on the three substrates pristane, biphenyl and anthracene. In each case, one strain was able to convert the isolation substrate as well as form metabolites (Fig. 3, Additional file 1: Tables S9, S12, S13). The genera *Leifsonia* was frequently mentioned in connection with growth on alkanes and aromatics [111], with the degradation of phenol [112], diketopiperazines [111] and insecticides [113] or with the occurrence in the microbiome of oil-contaminated soil [114]. However, *Leifsonia sp.* SBUG 2174, here described as pristane degrader, *Leifsonia* sp. SBUG 2175, a biphenyl converter, and *Leifsonia shinshuensis* SBUG 2180, an anthracene transformer, extend the scope of genus *Leifsonia* for crude oil component degradation.

The majority of the 110 isolated and characterized strains of 45 different genera belong to well-described hydrocarbon degraders like Bacilli and Rhodococci as well as to *Achromobacter*, *Gordonia*, *Pseudomonas*, *Stenotrophomonas*, *Aspergillus*, *Exophiala*, *Fusarium*, *Meyerozyma*, *Penicillium* and *Trichoderma* species. Strains from these genera have been isolated from polluted areas and investigated for decades [55, 62, 63, 70, 93, 115–122]. With all of these results on the metabolization of the used oil components we confirm their importance for the degradative power of the microbiome in polluted soils by showing that they are able to degrade the isolation substrates.

## Conclusions

In summary, it can be stated that starting from the microbiome of oil-contaminated soils, through the enrichment and isolation of pure cultures to the transformation of individual oil components by the isolates, a long methodological path has to be covered with numerous losses in diversity. But in the end, a comprehensive picture of the degradation of crude oil components emerges. This study highlights the selective enrichment of rare microbes specialised in degradation of various oil compounds, and the significance of both bacteria and fungi for bioremediation. Furthermore, it illustrates functional redundancy among the diverse soil microbes for utilization of individual aromatic and aliphatic compounds. Our study showed great potential of the isolated microbes and the indigenous microbiota in degrading oil, thus these results could be transferred to a large scale of in situ bioremediation. On the one hand, isolated oil-degrading bacteria and fungi can be used to assemble specialized consortia of hydrocarbon-degrading microorganisms, which are then applied to contaminated sites to support the indigenous communities during degradation of oil contamination of Kazakh soils. On the other hand, the indigenous microbial communities can also be supported by targeted measures such as the addition of trace or limited nutrients or targeted aeration of the soil.

## Supplementary Information

Below is the link to the electronic supplementary material.

Additional file 1

## Data Availability

The data generated or analysed during this study are included in this published article and its Additional file 1. The Illumina datasets generated and analysed during the current study are available in the European Nucleotide Archive repository, https://www.ebi.ac.uk/ena/browser/view/PRJEB87402.
