# Supplementary material for: The hydrocarbon-degrading bacteria and fungi in oil contaminated soils of Kazakhstan: microbiome composition, enrichment, isolation and bioremediation potential
Source: Environ Microbiome. 2026 Mar 7;21:49. doi: 10.1186/s40793-026-00866-y (PMC13064327; doi:10.1186/s40793-026-00866-y)
Supplement: Supplementary file 1 — Additional file 1 [file 40793_2026_866_MOESM1_ESM.pdf]

The hydrocarbon-degrading bacteria and fungi in oil contaminated soils of Kazakhstan – microbiome composition, enrichment, isolation and bioremediation potential

Felix Müller,[1,A] Haitao Wang,[1,A] Anne Reinhard,[1] Anel Omirbekova,[2] Ramza Berzhanova,[2] Togzhan Mukasheva,[2] Tim Urich,[1] Annett Mikolasch[1]*

[1] University Greifswald, Institute of Microbiology, Felix-Hausdorff-Straße 8, 17489 Greifswald, Germany

[2] Al-Farabi Kazakh National University, Department of Biology and Biotechnology, Al-Farabi Ave 71, 050040 Almaty, Kazakhstan

[A] Shared first authorship

* Corresponding author

Phone: ++49-3834-4205917

Fax: ++49-3834-4205902

Email: annett.mikolasch@uni-greifswald.de

Table S1 Sampling sites, sampling conditions**,** crude oil characteristics of contaminated soils in Kazakhstan

Sample	B1	B2	B3	BK
Region	Uzen oil deposit, Mangystau region	Uzen oil deposit, Mangystau region	Aktobe oil deposit, Kenkiyak region	park close to Almaty-1 train station
Longitude	52°46′56″ E	52°46′56″ E	57°05'11.0" E	76°56'30.7" E
Latitude	43°26′36″ N	43°26′36″ N	50°20'38.2" N	43°20'36.8" N
Sampling date	2018-01-15	2018-01-15	2018-04-24	2018-04-19
Soil salinity, %	3-5	3-5	7-9	0.5
Soil pH	8.5	8.5	9.0-9.3	5.1
Na^+, mmol/100 g soil	17.8	17.8	9,7	–
K^+, mmol/100 g soil	–	–	0,3	–
Mg^{2+}, mmol/100 g soil	2.80	2.80	3,7	–
Ca^{2+}, mmol/100 g soil	29.0	29.0	6,5	–
Cl^-, mmol/100 g soil	7.70	7.70	3,4	–
HCO_3^-, mmol/100 g soil	1.30	1.30	0,5	–
SO_4^{-2}, mmol/100 g soil	14.0	14.0	4,5	–
Contamination				
Contamination origin	Oil industry	Oil industry	Oil industry	Petroleum products spill
Residual hydrocarbon content (mg/kg)	60,000 – 80,000	100,000 – 250,000	10,000 – 30,000	500 – 1,000
Contaminating crude oil physical characteristics and composition				
Oil density, kg/m^3	844-874	844-874	821-850	–
Oil viscosity	high	high	low	–
Sulfur content, %	0.16-2.00	0.16-2.00	0.24-1.24	–
Paraffins, %	16.0-22.0	16.0-22.0	1.53-6.76	–
Silica gel resins, %	8.00-20.0	8.00-20.0	1.20-8.50	–
Asphaltenes, %	3.10-4.20	3.10-4.20	–	–
Naphthene-aromatic hydrocarbons and heterocyclic compounds, %	Round 40-50	Round 40-50	Round 80-90	-

Table S2 Number of microorganisms recovered from four different contaminated soils in Kazakhstan on eight different isolation substrates

**Substrate**	**Soil sample**	**MSMB**	**MSMF**	**Sum of isolated strains**
Kazakh crude oil	B1	-	1	1
	B2	-	1	1
	B3	10	3	13
	BK	9	3	12
	Sum	19	8	27
Tetradecane	B1	-	7	7
	B2	1	3	4
	B3	4	11	15
	BK	3	7	10
	Sum	8	28	36
Pristane	B1	2	-	2
	B2	-	-	-
	B3	3	2	5
	BK	4	8	12
	Sum	9	10	19
Heptamethylnonane	B1	-	7	7
	B2	-	-	-
	B3	nt[a)]	nt	
	BK	nt	nt	
	Sum	-	7	7
Cyclohexanone	B1	-	5	5
	B2	-	5	5
	B3	8	2	10
	BK	10	2	12
	Sum	18	14	32
Phenol	B1	-	1	1
	B2	-	3	3
	B3	-	4	4
	BK	4	-	4
	Sum	4	8	12
Biphenyl	B1	1	1	2
	B2	-	1	1
	B3	4	5	9
	BK	4	3	7
	Sum	9	10	19
Anthracene	B1	-	3	3
	B2	-	-	-
	B3	6	2	8
	BK	4	2	6
	Sum	10	7	17
Sum of each medium		77	92	
Sum of all microorganisms isolated as pure cultures				169

a) nt = not tested

Table S3 Relative abundance of Prokaryotes and Eukaryotes at the phylum/division level [%][a)] of contaminated soils in Kazakhstan

Prokaryotes				Eukaryotes			
Phylum	B1	B3	BK	Division	B1	B3	BK
Acidobacteria	0.009	4.872	21.053	*Ascomycota*	40.206	5.187	12.161
Actinobacteria	2.629	29.132	16.908	*Basidiomycota*	59.768	1.029	1.796
Bacteroidetes	1.191	2.621	5.955	*Cercozoa*	0.000	28.575	29.474
Chloroflexi	0.095	16.969	4.216	*Chlorophyta*	0.000	21.425	0.164
Firmicutes	10.381	5.688	0.174	*Ciliophora*	0.000	8.342	9.146
Gemmatimonadetes	0.025	0.303	3.561	*Mucoromycota*	0.000	0.271	1.463
Planctomycetes	0.068	0.254	4.841	*Nematoda*	0.000	0.081	26.989
Proteobacteria	85.432	24.386	36.781	*Ochrophyta*	0.000	12.216	6.862
Thaumarchaeota	0.038	0.041	3.133	*Phragmoplastophyta*	0.016	2.898	0.455
Verrucomicrobia	0.032	0.036	2.007	*Rotifera*	0.000	7.042	0.168
others[b)]	0.1	15.698	1.371	others	0.01	12.934	11.322

[a)] Values in which at least one of the soils achieved a percentage value greater than 1

[b)] Values in which all phyla/divisions achieved a percentage value smaller than 1

Table S4 Relative abundance of Prokaryotes and Eukaryotes at the class level [%][a)] of contaminated soils in Kazakhstan

Prokaryotes

Class	Phylum	B1	B3	BK
Acidobacteriia	*Acidobacteria*	0.005	0.748	1.342
Blastocatellia (Subgroup 4)		0.000	0.038	11.295
Holophagae		0.000	3.553	1.320
Subgroup 6		0.000	0.528	6.120
Acidimicrobiia	*Actinobacteria*	0.075	3.418	0.667
Actinobacteria		2.423	24.712	7.897
Thermoleophilia		0.097	0.922	7.838
Bacteroidia	*Bacteroidetes*	1.191	2.618	5.793
Anaerolineae	*Chloroflexi*	0.007	4.716	0.847
Chloroflexia		0.034	4.497	1.526
Gitt-GS-136		0.000	7.311	0.612
Bacilli	*Firmicutes*	10.324	0.049	0.127
Clostridia		0.027	5.634	0.046
Gemmatimonadetes	*Gemmatimonadetes*	0.020	0.266	2.644
Phycisphaerae	*Planctomycetes*	0.009	0.014	2.353
Planctomycetacia		0.059	0.241	1.987
Alphaproteobacteria	*Proteobacteria*	1.377	6.458	11.380
Deltaproteobacteria		0.032	0.281	2.668
Gammaproteobacteria		84.023	17.646	22.721
Nitrososphaeria	*Thaumarchaeota*	0.038	0.041	3.133
Verrucomicrobiae	*Verrucomicrobia*	0.032	0.036	2.007
unclassified	unclassified	0.011	15.519	0.117
others[b)]		0.216	0.754	5.56

[a)] Values in which at least one of the soils achieved a percentage value greater than 1

[b)] Values in which all classes achieved a percentage value smaller than 1

Eukaryotes

Class	Division	B1	B3	BK
Dothideomycetes	*Ascomycota* [b)]	39.585	0.190	2.634
Eurotiomycetes		0.413	2.045	0.262
Saccharomycetes		0.090	2.045	0.000
Sordariomycetes		0.032	0.731	8.620
Agaricomycetes	*Basidiomycota* [b)]	0.000	0.000	1.207
Microbotryomycetes		59.322	0.352	0.110
Cercomonadidae	*Cercozoa*	0.000	0.000	1.579
Phytomyxea		0.000	0.000	13.661
Thecofilosea		0.000	0.000	2.121
Incertae Sedis		0.000	2.478	2.443
unclassified		0.000	25.447	8.971
Chlorophyceae	*Chlorophyta*	0.000	14.653	0.148
Trebouxiophyceae		0.000	6.771	0.016
Intramacronucleata	*Ciliophora*	0.000	8.342	9.072
Incertae Sedis	*Mucoromycota* [b)]	0.000	0.271	1.003
Chromadorea	*Nematoda*	0.000	0.081	21.316
Enoplea		0.000	0.000	5.673
Chrysophyceae	*Ochrophyta*	0.000	8.220	6.755
Diatomea		0.000	1.287	0.054
Xanthophyceae		0.000	2.397	0.052
Embryophyta	*Phragmoplastophyta*	0.016	2.898	0.439
Bdelloidea	*Rotifera*	0.000	7.042	0.000
unclassified	unclassified	0.000	12.067	8.517
others[c)]		0.542	2.683	5.347

[a)] Values in which at least one of the soils achieved a percentage value greater than 1

[b)] kingdom fungi

[c)] Values in which all classes achieved a percentage value smaller than 1

Table S5 Relative abundance of Prokaryotes and Eukaryotes at the order level [%][a)] of contaminated soils in Kazakhstan

Prokaryotes

Order	Class	Phylum	B1	B3	BK
Pyrinomonadales	Blastocatellia (Subgroup 4)	*Acidobacteria*	0.000	0.004	7.301
Subgroup 7	*Holophagae*		0.000	3.553	1.320
unclassified	Subgroup 6		0.000	0.462	5.963
Corynebacteriales	*Actinobacteria*	*Actinobacteria*	1.461	6.734	0.253
Micrococcales			0.627	8.893	1.401
Propionibacteriales			0.009	3.813	0.550
Solirubrobacterales	*Thermoleophilia*		0.029	0.452	7.222
Chitinophagales	*Bacteroidia*	*Bacteroidetes*	0.027	0.020	3.367
Thermomicrobiales	*Chloroflexia*	*Chloroflexi*	0.034	4.457	1.207
unclassified	Gitt-GS-136		0.000	7.311	0.612
Lactobacillales	*Bacilli*	*Firmicutes*	10.286	0.000	0.000
Thermoanaerobacterales	*Clostridia*		0.000	3.955	0.000
Azospirillales	*Alphaproteobacteria*	*Proteobacteria*	0.009	0.398	3.238
Acidithiobacillales	*Gammaproteobacteria*		0.000	13.858	0.012
Betaproteobacteriales			0.953	0.972	20.047
Pseudomonadales			82.649	0.037	0.042
Nitrososphaerales	*Nitrososphaeria*	*Thaumarchaeota*	0.023	0.041	3.133
unclassified	unclassified	unclassified	0.011	15.519	0.117
others[b)]			3.882	29.521	44.215

[a)] Values in which at least one of the soils achieved a percentage value greater than 3

[b)] Values in which all orders achieved a percentage value smaller than 3

Eukaryotes

Order	Class	Division	B1	B3	BK
Pleosporales	*Dothideomycetes*	*Ascomycota*[b)]	39.585	0.000	1.566
Hypocreales	*Sordariomycetes*		0.032	0.081	7.176
Sporidiobolales	*Microbotryomycetes*	*Basidiomycota*[b)]	59.322	0.217	0.000
Phytomyxea	*Phytomyxea*	*Cercozoa*	0.000	0.000	13.661
unclassified	unclassified		0.000	25.447	8.971
Trebouxiales	*Trebouxiophyceae*	*Chlorophyta*	0.000	6.582	0.000
unclassified	*Chlorophyceae*		0.000	11.376	0.132
Spirotrichea	*Intramacronucleata*	*Ciliophora*	0.000	6.961	5.203
Tylenchida	*Chromadorea*	*Nematoda*	0.000	0.081	20.653
Triplonchida	*Enoplea*		0.000	0.000	5.673
Chromulinales	*Chrysophyceae*	*Ochrophyta*	0.000	6.419	5.305
Adinetida	*Bdelloidea*	*Rotifera*	0.000	7.042	0.000
unclassified	unclassified	unclassified	0.000	12.067	8.517
others[c)]			1.061	23.727	23.143

a) Values in which at least one of the soils achieved a percentage value greater than 3

b) kingdom fungi

c) Values in which all orders achieved a percentage value smaller than 3

Table S6 Relative abundance of Prokaryotes and Eukaryotes at the family level [%][a)] of contaminated soils in Kazakhstan

Prokaryotes

Family	Order	Class	Phylum	B1	B3	BK
Pyrinomonadaceae	*Pyrinomonadales*	Blastocatellia (Subgroup 4)	*Acidobacteria*	0.000	0.004	7.301
unclassified	Subgroup 7	*Holophagae*		0.000	3.553	1.320
unclassified	unclassified	Subgroup 6		0.000	0.462	5.963
Mycobacteriaceae	*Corynebacteriales*	*Actinobacteria*	*Actinobacteria*	0.233	5.708	0.253
Microbacteriaceae	*Micrococcales*			0.165	5.020	0.285
Nocardioidaceae	*Propionibacteriales*			0.009	3.813	0.550
67-14	*Solirubrobacterales*	*Thermoleophilia*		0.020	0.322	5.085
JG30-KF-CM45	*Thermomicrobiales*	*Chloroflexia*	*Chloroflexi*	0.034	4.453	1.132
unclassified	unclassified	Gitt-GS-136		0.000	7.311	0.612
Carnobacteriaceae	*Lactobacillales*	*Bacilli*	*Firmicutes*	9.323	0.000	0.000
Thermoanaerobacteraceae	*Thermoanaerobacterales*	*Clostridia*		0.000	3.531	0.000
Acidithiobacillaceae	*Acidithiobacillales*	*Gammaproteobacteria*	*Proteobacteria*	0.000	13.858	0.012
Methylophilaceae	*Betaproteobacteriales*			0.000	0.000	13.047
Pseudomonadaceae	*Pseudomonadales*			82.547	0.028	0.042
Nitrososphaeraceae	*Nitrososphaerales*	*Nitrososphaeria*	*Thaumarchaeota*	0.023	0.041	3.133
unclassified	unclassified	unclassified	unclassified	0.011	15.519	0.117
others[b)]				7.635	36.377	61.148

[a)] Values in which at least one of the soils achieved a percentage value greater than 3

[b)] Values in which all families achieved a percentage value smaller than 3

Eukaryotes

Family	Order	Class	Division	B1	B3	BK
Leptosphaeriaceae	*Pleosporales*	*Dothideomycetes*	*Ascomycota*[b)]	13.169	0.000	1.097
Pleosporaceae				26.391	0.000	0.351
Nectriaceae	*Hypocreales*	*Sordariomycetes*		0.032	0.000	6.737
Sporidiobolaceae	*Sporidiobolales*	*Microbotryomycetes*	*Basidiomycota*[b)]	59.322	0.217	0.000
Phytomyxea	*Phytomyxea*	*Phytomyxea*	*Cercozoa*	0.000	0.000	13.661
unclassified	unclassified	unclassified		0.000	25.447	8.971
Trebouxiales	*Trebouxiales*	*Trebouxiophyceae*	*Chlorophyta*	0.000	6.582	0.000
unclassified	unclassified	*Chlorophyceae*		0.000	11.376	0.132
Hypotrichia	*Spirotrichea*	*Intramacronucleata*	*Ciliophora*	0.000	6.961	5.203
unclassified	*Tylenchida*	*Chromadorea*	*Nematoda*	0.000	0.081	20.653
unclassified	*Triplonchida*	*Enoplea*		0.000	0.000	5.673
Chromulinales	*Chromulinales*	*Chrysophyceae*	*Ochrophyta*	0.000	6.419	5.305
unclassified	*Adinetida*	*Bdelloidea*	*Rotifera*	0.000	7.042	0.000
unclassified	unclassified	unclassified	unclassified	0.000	12.067	8.517
others[c)]				1.086	23.808	23.7

[a)] Values in which at least one of the soils achieved a percentage value greater than 3

[b)] kingdom fungi

[c)] Values in which all families achieved a percentage value smaller than 3

Table S7 Relative abundance of Prokaryotes and Eukaryotes at the genus level [%][a)] of contaminated soils in Kazakhstan

Prokaryotes

Genus	Family	Order	Class	Phylum	B1	B3	BK
RB41	*Pyrinomonadaceae*	*Pyrinomonadales*	Blastocatellia (Subgroup 4)	*Acidobacteria*	0.000	0.004	7.301
unclassified	unclassified	Subgroup 7	*Holophagae*		0.000	3.553	1.320
unclassified	unclassified	unclassified	Subgroup 6		0.000	0.462	5.963
Mycobacterium	*Mycobacteriaceae*	*Corynebacteriales*	*Actinobacteria*	*Actinobacteria*	0.233	5.708	0.253
Nocardioides	*Nocardioidaceae*	*Propionibacteriales*			0.009	3.556	0.267
unclassified	67-14	*Solirubrobacterales*	*Thermoleophilia*		0.020	0.322	5.085
unclassified	JG30-KF-CM45	*Thermomicrobiales*	*Chloroflexia*	*Chloroflexi*	0.034	4.453	1.132
unclassified	unclassified	unclassified	Gitt-GS-136		0.000	7.311	0.612
Carnobacterium	*Carnobacteriaceae*	*Lactobacillales*	*Bacilli*	*Firmicutes*	7.881	0.000	0.000
Caloribacterium	*Thermoanaero-bacteraceae*	*Thermoanaero-bacterales*	*Clostridia*		0.000	3.531	0.000
KCM-B-112	*Acidithio-bacillaceae*	*Acidithiobacillales*	*Gammaproteo-bacteria*	*Proteobacteria*	0.000	13.858	0.012
Methylotenera	*Methylophilaceae*	*Betaproteo-bacteriales*			0.000	0.000	13.013
Pseudomonas	*Pseudomonadaceae*	*Pseudomonadales*			82.547	0.028	0.042
unclassified	unclassified	unclassified	unclassified	unclassified	0.011	15.519	0.117
others[b)]					9.265	41.695	64.883

[a)] Values in which at least one of the soils achieved a percentage value greater than 3

[b)] Values in which all genera achieved a percentage value smaller than 3

Eukaryotes

Genus	Family	Order	Class	Division	B1	B3	BK
Neophaeosphaeria	*Leptosphaeriaceae*	*Pleosporales*	*Dothideomycetes*	*Ascomycota*[b)]	13.169	0.000	1.097
Alternaria	*Pleosporaceae*				23.752	0.000	0.000
Fusarium	*Nectriaceae*	*Hypocreales*	*Sordariomycetes*		0.032	0.000	6.737
unclassified	*Sporidiobolaceae*	*Sporidiobolales*	*Microbotryomycetes*	*Basidiomycota*[b)]	59.322	0.217	0.000
Plasmodiophora	*Phytomyxea*	*Phytomyxea*	*Phytomyxea*	*Cercozoa*	0.000	0.000	13.661
unclassified	unclassified	unclassified	unclassified		0.000	25.447	8.971
Trebouxia	*Trebouxiales*	*Trebouxiales*	*Trebouxiophyceae*	*Chlorophyta*	0.000	6.582	0.000
unclassified	unclassified	unclassified	*Chlorophyceae*		0.000	11.376	0.132
unclassified	*Hypotrichia*	*Spirotrichea*	*Intramacronucleata*	*Ciliophora*	0.000	4.740	3.901
unclassified	unclassified	*Tylenchida*	*Chromadorea*	*Nematoda*	0.000	0.081	20.653
unclassified	unclassified	*Triplonchida*	*Enoplea*		0.000	0.000	5.673
Spumella	*Chromulinales*	*Chromulinales*	*Chrysophyceae*	*Ochrophyta*	0.000	6.419	5.305
unclassified	unclassified	*Adinetida*	*Bdelloidea*	*Rotifera*	0.000	7.042	0.000
unclassified	unclassified	unclassified	unclassified	unclassified	0.000	12.067	8.517
others[c)]					3.725	26.029	25.353

[a)] Values in which at least one of the soils achieved a percentage value greater than 3
[b)] kingdom fungi
[c)] Values in which all genera achieved a percentage value smaller than 3

Table S8 Transformation experiments on the substrate tetradecane with pure cultures of isolated strains from contaminated soils in Kazakhstan

**Strain**	**Estimated substrate reduction [%]**	**Detected products**		
	**Tetradecane**	**Mono-carboxylic acids**	**Dicarboxylic acids**	**Ketones**
Strains isolated on tetradecane				
Prokaryotes				
Achromobacter mucicolens SBUG 2101	73.7±10.6	+	-	-
Brevibacillus sp. SBUG 2104	~ 0	+	-	+ (6-tetradecanone)
Brucella intermedia SBUG 2121	~ 0	+	-	(+) (6-tetradecanone)
Caballeronia sp. SBUG 2148	~ 0	+	-	-
Neobacillus drentensis SBUG 2131, 2132	~ 0	+	-	-
Paraburkholderia graminis SBUG 2133	~ 0	+	-	(+) (6-tetradecanone)
Psychrobacillus sp. SBUG 2324	~ 0	+	-	+ (6-tetradecanone)
Rhodococcus qingshengii SBUG 2120	~ 0	(+)	-	-
Serratia plymuthica SBUG 2102	69.2±11.2	+	-	-
Xanthobacter sp. SBUG 2113	~ 0	+	-	+ (6-dodecanone)
Eukaryotes				
Aspergillus sp. SBUG-M 1743	79.6±11.6	(+)	-	-
Cystobasidium slooffiae SBUG-Y 2206	99.4±1.6	+	-	-
Exophiala phaeomuriformis SBUG-Y 2222	~ 0	+	-	-
Exophiala sp. SBUG-Y 2223	~ 0	(+)	-	-
Meyerozyma guilliermondii SBUG-Y 2205, 2209, 2210, 2212, 2218	90.5±12.3	+	+	-
Penicillium javanicum SBUG-M 1741, 1742, 1744	97.7±0.6	+	-	-
Sarocladium sp. SBUG-Y 2208	44.6±10.6	+	-	+ (tetradecanone)
Scedosporium boydii SBUG-M 1749	40.9±5.5	+	+	-
Trichoderma harzianum SBUG-M 1750, 1771	51.5±9.2	+	-	-
Strains isolated on crude oil but cultivated on tetradecane				
Prokaryotes				
Achromobacter insolitus SBUG 2143, 2144	93.6±0.4	-	-	+ (6-tetradecanone)
Amycolatopsis sp. SBUG 2149	84.4±22.1	+	-	-
Microbacterium sp. SBUG 2123	~ 0	+	-	-
Peribacillus frigoritolerans SBUG 2142	57.5±14.5	+	-	(+) (6-dodecanone)<br>(+) (6-tetradecanone)
Peribacillus frigoritolerans SBUG 2150	71.7±4.5	-	-	(+) (6-tetradecanone)
Peribacillus sp. SBUG 2118	20.0±11.5	(+)	-	-
Peribacillus sp. SBUG 2128	56.2±14.5	+	-	-

Xanthobacter sp. SBUG 2115	54.3±3.5	+	-	(+) (2-tetradecanone)
Eukaryotes				
Fusarium oxysporum SBUG-M 1768, 1769	84.9±8.9	+	-	-
Meyerozyma guilliermondii SBUG-Y 2219	5.7±1.5	+	+	-
Penicillium javanicum SBUG-M 1770	99.9±0.1	+	-	-

Table S9 Transformation experiments on the substrate pristane with pure cultures of isolated strains from contaminated soils in Kazakhstan

Strain	Estimated substrate reduction [%]	Detected products		
	Pristane	Branched chain monocarboxylic acids	Branched chain dicarboxylic acids	Ketones
Strains isolated on pristane				
Prokaryotes				
Achromobacter spanius SBUG 2140	~ 0	-	-	-
Amycolatopsis sp. SBUG 2106	~ 0	-	-	-
Bordetella sp. SBUG 2340	~ 0	-	-	-
Leifsonia sp. SBUG 2139, 2146	~ 0	-	-	-
Leifsonia sp. SBUG 2174	55.6±32.7	-	+	-
Inquilinus sp. SBUG 2141	~ 0	-	-	-
Massilia sp. SBUG 2145	~ 0	-	-	-
Micrococcus luteus SBUG 2138	~ 0	-	-	-
Paraburkholderia graminis SBUG 2103	~ 0	-	-	-
Rhodanobacter sp. SBUG 2107	~ 0	-	-	-
Staphylococcus epidermidis SBUG 2168	10.0±5.4	-	+	-
Eukaryotes				
Purpureocillium lilacinum SBUG-M 1751	99.5±0.1	-	+	-
Strains isolated on crude oil but cultivated on pristane				
Eukaryotes				
Fusarium oxysporum SBUG-M 1747	95.8±1.6	-	+	-

Table S10 Transformation experiments on the substrate cyclohexanone with pure cultures of isolated strains from contaminated soils in Kazakhstan

Strain	Estimated substrate reduction [%]	Detected products		
	Cyclohexanone	Cyclo-hexanols	ε-Caprolactone	Hexanedioic acid
Strains isolated on cyclohexanone				
Prokaryotes				
Chryseobacterium sp. SBUG 2116	82.3±18.2	+	+	+
Georgenia sp. SBUG 2109	~ 0	+	-	-
Georgenia sp. SBUG 2112	62.5±10.6	+	(+)	(+)
Gottfriedia sp. SBUG 2119	~ 0	+	+	+
Microbacterium sp. SBUG 2108, 2117	~ 0	+	+	-
Microbacterium sp. SBUG 2124	18.54±4.7	+	+	(+)
Neorhizobium petrolearium SBUG 2169	100±0.2	+	-	+
Peribacillus frigoritolerans SBUG 2130	61.8±24.8	+	-	+
Peribacillus frigoritolerans SBUG 2136	83.0±13.7	(+)	+	-
Eukaryotes				
Fusarium oxysporum SBUG-M 1746	88.4±4.6	+	+	+
Fusarium oxysporum SBUG-M 1748	98.1±0.1	+	+	+
Meyerozyma guilliermondii SBUG-Y 2207	4.1±2.6	+	-	+
Rhodotorula mucilaginosa SBUG-Y 2211	93.8±5.6	+	-	-
Rhodotorula mucilaginosa SBUG-Y 2224	98.8±1.6	+	+	(+)
Strains isolated on crude oil but cultivated on cyclohexanone				
Prokaryotes				
Georgenia sp. SBUG 2122 (P8K1.2)	~ 0	+	(+)	-

Table S11 Transformation experiments on the substrate phenol with pure cultures of isolated strains from contaminated soils in Kazakhstan

**Strain**	**Estimated substrate reduction [%]**	**Detected products**			
	**Phenol**	**Muconic acids**	**Quinones**	**Unknown products [a)]**	**Dimers (186 g/mol)**
Strains isolated on phenol					
Prokaryotes					
Gordonia rubripertincta SBUG 2151	100±0.1	-	-	-	(+) (dihydroxybiphenyl)
Peribacillus frigoritolerans SBUG 2160, 2161, 2162	~ 0	-	-	-	-
Peribacillus simplex SBUG 2164	~ 0	-	+ (*o*-hydro-quinone)	+ (5)	+ (4-phenoxyphenol, 2-(2-methoxyphenyl)phenol)
Pseudarthrobacter oxydans SBUG 2147	100±0.3	+ (2)	-	+ (6)	-
Eukaryotes					
Meyerozyma guilliermondii SBUG-Y 2221	100±0.1	-	+ (*p*-hydro-quinone, *p*-benzo-quinone)	-	+ (4,4'-dihydroxybiphenyl, 4-phenoxyphenol, 3,3'-dihydroxybiphenyl, 2-phenoxyphenol
Strains isolated on crude oil but cultivated on phenol					
Eukaryotes					
Meyerozyma guilliermondii SBUG-Y 2214	100±0.4	-	+ (*p*-hydro-quinone, *p*-benzo-quinone)	-	+ (4,4'-dihydroxybiphenyl, 4-phenoxyphenol, 3,3'-dihydroxybiphenyl, 2-phenoxyphenol

[a)] number in brackets = number of products

Table S12 Transformation experiments on the substrate biphenyl with pure cultures of isolated strains from contaminated soils in Kazakhstan

Strain[a)]	Detected products			
	**Monohydroxylated biphenyls (OHBP)**	**Dihydroxylated biphenyls (DiOHBP)**	**Ring fission products[b)]**	**Unknown products[b)]**
Prokaryotes				
Bordetella sp. SBUG 2134, 2135	-	-	-	-
Caballeronia sp. SBUG 2114	-	-	-	-
Cellulosimicrobium cellulans SBUG 2163	-	-	-	-
Leifsonia sp. SBUG 2175	+ (2-, 4-OHBP)	-	-	+ (4)
Microbacterium sp. SBUG 2176	+ (2-OHBP)	-	-	+ (7)
Paenibacillus sp. SBUG 2166	-	-	-	+ (1)
Peribacillus frigoritolerans SBUG 2137	-	-	-	+ (1)
Priestia aryabhattai SBUG 2155	+ (2-, 4-OHBP)	+ (3,4-, 2,5-DiOHBP)	+ (2)	+ (1)
Priestia megaterium SBUG 2165	+ (2-, 3-, 4-OHBP)	+ (2,3-, 3,4-, 2,5-, 4,4‘-DiOHBP,	+ (2)	+ (1)
Rhodococcus erythropolis SBUG 2156	+ (2-, 4-OHBP)	+ (2,2‘-, 4,4‘-DiOHBP)	-	-
Eukaryotes				
Meyerozyma guilliermondii SBUG-Y 2216, 2220	+ (2-, 3-, 4-OHBP)	-	-	-
Strains isolated on crude oil but cultivated on biphenyl				
Prokaryotes				
Roseococcus sp. SBUG 2182	-	-	-	-

a) Estimated substrate reduction could not be screened because of the low water solubility of biphenyl

b) number in brackets = number of products

Table S13 Transformation experiments on the substrate anthracene with pure cultures of isolated strains from contaminated soils in Kazakhstan

**Stain**[a)]	**Detected products**				
	**Anthrone**	**9,10-Anthracendione**	**3-Hydroxy-2-naphthoic acid**	**Muconic acids**	**Unknown products**[b)]
Strains isolated on anthracene					
Prokaryotes					
Arthrobacter sp. SBUG 2126	-	+	-	-	-
Caballeronia sp. SBUG 2159	-	+	-	-	+ (5)
Cupriavidus alkaliphilus SBUG 2170	+	+	-	-	+ (9)
Leifsonia shinshuensis SBUG 2180	+	+	-	-	+ (3)
Priestia megaterium SBUG 2154	-	-	-	-	-
Pseudarthrobacter oxydans SBUG 2167	-	-	-	+	+ (1)
Pseudarthrobacter siccitolerans SBUG 2110, 2127	-	-	-	-	-
Pseudarthrobacter sp. SBUG 2111	-	+	-	-	-
Pseudarthrobacter sp. SBUG 2158	-	-	-	-	+ (1)
Pseudomonas putida SBUG 2172	-	+	-	-	+ (5)
Pseudomonas putida SBUG 2179	+	+	-	-	+ (3)
Stenotrophomonas acidaminiphila SBUG 2129	-	-	-	-	-
Stenotrophomonas sp. SBUG 2171	-	(+)	+	-	+ (8)
Eukaryotes					
Meyerozyma guilliermondii SBUG-Y 2217	-	+	-	-	+ (7)

[a)] Estimated substrate reduction could not be screened because of the low water solubility of anthracene
[b)] number in brackets = number of products
